# Mapping and predicting groundwater accumulations using remote sensing and aeromagnetic data: a case study from Bahariya Oasis, Western Desert, Egypt

**DOI:** 10.1038/s41598-026-42907-z

**Published:** 2026-03-26

**Authors:** Hussein T. El-Badrawy, Saif M. Abo Khashaba, Sultan A. S. Araffa, Moamena Kassab, Mostafa Nagy

**Affiliations:** 1 Geology Department, Faculty of Science, Kafr Elsheikh University, Kafr Elsheikh, Egypt; 2 National Research Institute of Astronomy and Geophysics (NRIAG), Helwan, 11421 Cairo Egypt

**Keywords:** Bahariya, Groundwater potentiality, AHP, GIS modeling, Aeromagnetic, RTP, Environmental sciences, Hydrology, Natural hazards, Solid Earth sciences

## Abstract

The Bahariya Oasis in Egypt’s Western Desert faces critical water security challenges due to intensive groundwater exploitation and limited renewable recharge. To address this challenge, this study integrates a GIS-AHP model for the first time in the Bahariya area and incorporates high-resolution aeromagnetic data to enhance the prediction of groundwater potential zones. Nine groundwater-controlling thematic layers were compiled from multi-source datasets, classified, and weighted using the AHP model: structural geology (lineament density), terrain (slope), hydrology (drainage density and rainfall), hydrogeology (lithology and soil moisture), land-surface conditions (NDVI and LU/LC), and the RTP aeromagnetic signal. The resulting groundwater potential map delineates coherent “very-high” potential corridors concentrated in the southern and southeastern sectors, where high lineament density, low-relief topography, and permeable Quaternary alluvial deposits coincide and correlate with known productive wells, suggesting favorable conditions for enhanced recharge and storage. Potential patterns also show strong spatial correlation with the drainage network and mapped structural trends. Model validation against well occurrences yields an AUC of 93.4%, indicating excellent predictive accuracy. Aeromagnetic interpretation further resolves the subsurface structural architecture and constrains the basement configuration, revealing a marked deepening of the basement toward the south and southeast. This is where a thick sedimentary cover suggests enhanced groundwater storage potential, providing additional support for the groundwater potential zoning results. This integrated workflow offers a robust tool for groundwater prediction and planning, with direct utility for prioritizing drilling locations and supporting sustainable water resources planning in arid environments.

**Supplementary Information:**

The online version contains supplementary material available at 10.1038/s41598-026-42907-z.

## Introduction

Water is a fundamental necessity for human survival and for supporting agricultural expansion, urban growth, and industrial activities. Situated in the northeastern part of Africa, Egypt lies within an arid desert belt where rainfall is scarce, and surface water resources are limited. The Nile River remains the country’s primary source of surface freshwater, flowing from south to north. In light of Egypt’s rapidly increasing population, intensified socioeconomic demands, and declining per-capita water availability, groundwater has become an increasingly vital resource. This reliance is further amplified by emerging challenges, most notably the projected effects of climate change and the hydrological consequences of the Grand Ethiopian Renaissance Dam, which together pose significant risks to the continuity and stability of Nile-derived freshwater supplies.

**Fig. 1:**
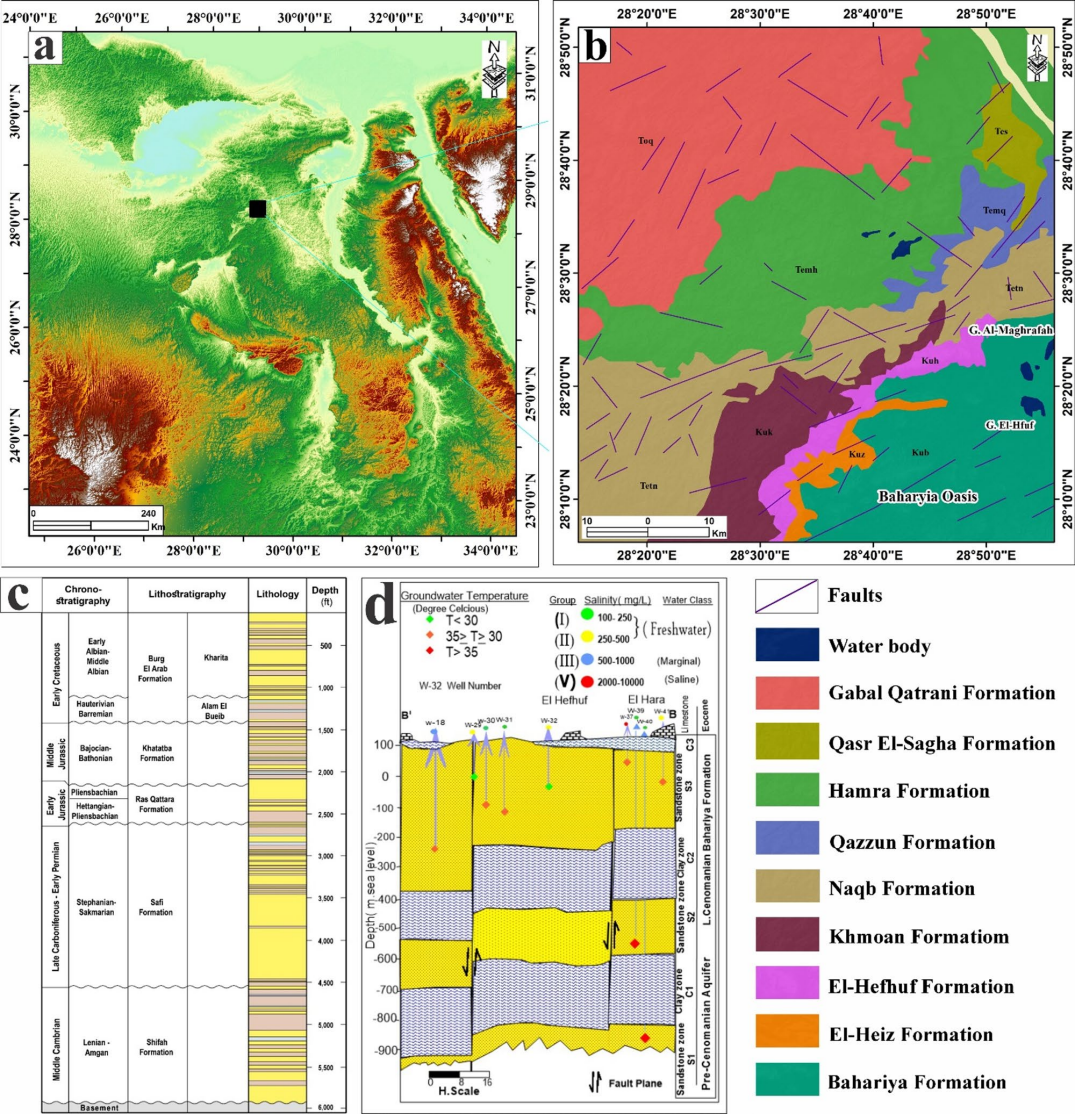
(a) Geologic map of the study area (after^4^. (C) The stratigraphic column for the Bahariya-1 well (after^5,6^. (D) The general hydrogeological cross-section of the Bahariya area (after^7^. The figure was created by ArcGIS Desktop 10.8. software; https://www.esri.com/en-us/arcgis/products/arcgis-desktop/overview.

Growing pressures on water resources have intensified the reliance on groundwater, making the assessment of groundwater availability more critical than ever. Understanding groundwater potential requires accurately identifying recharge pathways, whether derived from infiltrating surface water or from adjacent aquifers connected through structural features. The study area is situated within the Bahariya Oasis, a natural depression in the central Western Desert of Egypt, approximately 370 km southwest of Cairo (Fig. 1). Previous research has consistently emphasized the strategic importance of the Bahariya Oasis, noting its considerable economic and developmental promise^1–3^. In line with these findings, the Egyptian government has designated this zone as a priority location for major national initiatives spanning agriculture, industry, and urban development.

Previous groundwater studies in the Bahariya area have provided valuable hydrochemical, stratigraphic, and borehole-based insights; however, a clearly defined, regionally consistent geophysical framework remains lacking, creating critical gaps in coverage. In particular, earlier investigations show limited integration of magnetic and structural geophysical data, insufficient characterization of deep-seated faults and basement relief controlling aquifer geometry and hydraulic connectivity, and an absence of depth-constrained structural models capable of resolving subsurface fault continuity beyond surface mapping. Consequently, the role of basement architecture and fracture-controlled permeability in governing groundwater occurrence and recharge pathways has not been adequately addressed at the regional scale. The present study directly addresses this gap by integrating aeromagnetic interpretation, depth-estimation techniques, and available borehole information to provide a depth-constrained structural framework for groundwater exploration in the Bahariya depression. Recent research suggests that remote-sensing datasets can reliably support the delineation of groundwater-prospective zones, particularly when combined with multi-criteria decision-making frameworks such as the Analytic Hierarchy Process (AHP)^8–11^. The Analytical Hierarchy Process (AHP) is a well-established decision-support tool that organizes complex problems into a clear hierarchy and combines multiple evaluation criteria with expert judgment in a transparent way. Applied to groundwater potential mapping, an AHP-guided workflow enables practitioners to merge quantitative spatial evidence with informed professional insight, producing maps that are more defensible for exploration targeting and more useful for groundwater planning and management^10,11^. Although the AHP is widely used to derive factor weights in GIS-based groundwater potential susceptibility mapping, its results can be constrained by several well-documented methodological limitations.

AHP weights are ultimately based on expert judgement, so outcomes may reflect subjective preferences, expert selection, and local experience rather than measurable hydrologic causality, especially when empirical calibration data are limited^12,13^. The method can also be sensitive to how criteria are defined, scaled, and structured in the hierarchy; small changes in pairwise comparisons or in the set of alternatives/criteria may lead to different rankings, and the “rank reversal” phenomenon has been reported as a known concern in AHP applications^14,15^. Consequently, groundwater studies using AHP commonly recommend consistency checking (CR), sensitivity analysis, and, where possible, validation against well yields/groundwater levels or hybridization with data-driven models to reduce subjectivity and test robustness^12,16^. However, despite these limitations, AHP is still widely used for predicting groundwater potential zones^9–11^. Integrating AHP results with geophysical validation notably enhances the accuracy of the outcomes^9–17^, and can assist decision-makers in planning effective groundwater extraction strategies. In this study, we used 193 well-documented wells for validation.

Through the interpretation of remote-sensing imagery, researchers can identify and map a range of surface features that influence how runoff infiltrates the ground, including geological structures, drainage patterns, soil characteristics, and climatic conditions. Although these features are observable at the surface, they serve as valuable proxies for underlying hydrogeological properties, including groundwater recharge, potential for accumulation, and storage capacity^10,11,18^. When these surface indicators are examined in conjunction with high-resolution aeromagnetic data, particularly in delineating groundwater potential zones, the understanding of subsurface conditions becomes more robust and reliable^10,18^.

Magnetic investigations offer a fundamental geophysical basis for interpreting groundwater occurrence in structurally influenced basins by revealing basement morphology, fault systems, and areas of intensified fracturing. Aeromagnetic responses mainly arise from contrasts in magnetic susceptibility between crystalline basement rocks and weakly magnetic sedimentary cover, with anomaly wavelength and amplitude providing indirect constraints on basement topography that controls aquifer thickness and groundwater storage and flow. Faults and major structural discontinuities are identified through high-precision edge-detection analyses, which may act as conduits or barriers to flow. Irregular or heterogeneous magnetic patterns are commonly linked to fractured or altered basement domains and are interpreted as zones of enhanced secondary porosity and permeability, influencing groundwater recharge, storage, and hydraulic connectivity. Although magnetic data do not image groundwater directly, they supply critical structural and lithological information that, when integrated with geological and hydrogeological information, support a more physically robust evaluation of groundwater potential^19,20^. Recent improvements in instrumentation, low-altitude airborne platforms, and advanced processing techniques have greatly enhanced the resolution of aeromagnetic datasets, making them increasingly valuable for subsurface structural mapping. In groundwater studies, magnetic data provides essential insights during reconnaissance and detailed investigations alike. For example^10^, applied aeromagnetic analysis to identify the structural trends within the Sohag government, demonstrating how these features can influence groundwater distribution in the area^21^. employed aeromagnetic techniques to characterize structural trends in Wadi Qena that influence groundwater distribution. Similarly^19^, integrated magnetic and geoelectrical data to identify groundwater-bearing zones in Wadi Hagul.

Groundwater mapping and prediction across extensive areas is rarely straightforward when relying solely on field measurements, as access limitations and the sheer scale of the terrain can leave critical gaps in coverage. This study aims to detect the most favorable sites for groundwater reserves by delineating prospective groundwater occurrences and their likely recharge pathways. In addition, this study presents a novel groundwater potential map of the Bahariya Oasis, demonstrating for the first time how the integration of remote sensing and aeromagnetic data can be used to identify both surface and subsurface structural controls on groundwater accumulation and presents a transferable, data-efficient workflow suitable for data-scarce arid regions worldwide. By adopting this integrated approach, the study minimizes dependence on empirical borehole siting practices, ultimately improving the success rate of groundwater drilling operations. This coupled surface–subsurface approach provides a practical, region-scale tool for prioritizing high-potential targets for subsequent field verification and groundwater development.

## Geological and hydrological settings

The Bahariya Oasis, located in Egypt’s Western Desert about 370 km southwest of Cairo, exposes a stratigraphic succession spanning the Cretaceous to the Quaternary. This broad geological range offers a clear window into the region’s long-term depositional and environmental history. In the study area, the Cretaceous succession is commonly subdivided into the Bahariya, El-Heiz, El-Hefhuf, and Khoman (Chalk) formations, following the classic regional stratigraphic framework^22–24^. The Upper Eocene succession comprises the Qasr El-Sagha and Hamra formations, the Middle Eocene is represented by the Qazzun Formation, and the Lower Eocene corresponds to the Naqb Formation. Early Cenomanian deposits of the Bahariya Formation are unconformably overlain by Middle Eocene strata, which are subsequently covered by the Oligocene Gabal Qatrani Formation^23,25^. The sedimentary succession throughout the area is intruded by Tertiary basalt and dolerite bodies, which appear as sills and dykes that cut across the stratigraphy. Quaternary deposits cap the sequence and consist mainly of aeolian sands and lacustrine sediments, along with patches of recent continental sabkha deposits (Fig. 1b). In accordance with the Bahariya-1 well (Fig. 1c), the subsurface sequence starts with Precambrian basement, overlain unconformably by Paleozoic strata^5,6^: Middle Cambrian shale-rich beds followed by Late Carboniferous–Early Permian sandstones with minor shale. Above this, Mesozoic–Cenozoic rocks fall into four broad sedimentary phases: Jurassic clastics (Ras Qattara–Khatatba) capped by Masajid carbonates; Early Cretaceous mixed clastics (Alam El Bueib) ending with Alamein Dolomite and Dahab Shale; a mid-Cretaceous to early Tertiary package from Kharita–Bahariya into Abu Roash and Khoman, capped by Eocene Apollonia carbonates; and younger Dabaa–Moghra clastics overlain by Marmarica Limestone, with local thinning absence over structural highs. Stratigraphic succession is constrained by the spatial distribution of available wells and surface stratigraphic frameworks; therefore, lateral heterogeneity and local facies variations may not be fully resolved.

The study area is structurally controlled by three main fault sets with distinct strike directions. The first trend is oriented NE–SW, broadly parallel to the main Bahariya anticline, and exhibits throws that are commonly on the order of ~ 40–50 m (Fig. 1b). The second one, widely developed set trends NW–SE and typically displays slightly smaller displacement, with throws of about ~ 30–40 m compared to the NE–SW faults. The least frequent faults trend approximately E–W, although their throws can still be substantial, reaching ~ 40 m in places^6–28^.

Hydrologically, the Bahariya Oasis is an isolated desert system sustained almost entirely by groundwater^29^. It is situated within the northeastern part of the Nubian Sandstone Aquifer System (NSAS), one of the largest and most significant transboundary aquifers in North Africa. The depression’s faults and folds act like hidden walls, compartmentalizing the subsurface, redirecting flow, and sometimes providing vertical pathways that promote leakage or mixing between stratigraphic levels^30^. Alongside the deep regional system, Bahariya also commonly develops a shallow oasis groundwater source: a near-surface water table (often semi-confined or locally perched) in Quaternary–Recent sands/alluvium and depression-fill sediments, which is frequently maintained by irrigation return flow and seepage rather than by rainfall, making it the most responsive groundwater component in cultivated zones^2^. The primary groundwater supply is the Nubian sandstone aquifer, which consists of successive sandstone horizons interbedded with shale and clay, resulting in a multilayer, confined system that has historically supported artesian conditions in Western Desert oases^27,29^.

The groundwater-bearing formations are organized into three main hydrostratigraphic levels: a lower aquifer zone (S1), a middle zone (S2), and an upper zone (S3) (Fig. 1d). The system consists predominantly of continental sandstone sequences interlayered with clay and shale. These are separated by intervening layers of clay and shale that act as confining units. Its saturated thickness ranges widely, from roughly 100 m to more than 1500 m, and is further subdivided into two principal groundwater units: the Pre-Cenomanian and Cenomanian sub-aquifers^31^. The Pre-Cenomanian aquifer is composed mainly of coarse to very coarse sandstone with intercalated clay units. The Cenomanian aquifer includes the Bahariya and Heiz formations. The Bahariya Formation consists of successive sandstone beds interlayered with claystone and siltstone, whereas the Heiz Formation is characterized by dolomitic sandstone associated with calcareous clay. The aquifer system in the study area is hydraulically linked to adjacent and underlying groundwater-bearing formations through faults or permeable pathways that facilitate upward leakage^32^. Groundwater flow within the Nubian Sandstone Aquifer at the Bahariya Oasis is inherently complex, primarily governed by structural elements that create interconnected pathways and channels, facilitating upward leakage from deeper aquifer units.

Additionally, the Nubian Aquifer can be regarded as a multilayered system. The deepest zone (S1) exhibits the highest piezometric head in the study area, reaching approximately 152 m above sea level, whereas the shallowest zone (S3) records the lowest head, at about 88 m above sea level^3,27^. Groundwater in the Bahariya area generally flows from the southwest toward the northeast. In contrast, aquifer transmissivity and hydraulic conductivity decrease progressively in the same direction, primarily due to increasing shale and clay content within the NSA system^3,27^. The NSA System was historically characterized by artesian conditions, with naturally flowing wells prior to intensive development. Since the mid-20th century, agricultural expansion and increased groundwater abstraction have caused a sustained decline in artesian heads, shifting most wells to pumped conditions. These drawdown trends indicate growing aquifer stress and localized depletion, which reduce natural discharge and underscore the role of structurally controlled recharge zones in controlling present groundwater potential^4,27^.

## Data and methods

### Remote sensing data

To delineate potential groundwater zones within the Bahariya area, an integrated approach combining remote sensing imagery with GIS analysis was employed. The study incorporated several key datasets:

#### Radar data and terrain analysis

Surface lineaments were mapped using Sentinel-1B C-band synthetic aperture radar imagery, acquired on September 8, 2021. Structural features were automatically extracted using the LINE algorithm in Geomatica-PCI software, enabling identification of predominant fault and fracture trends. The orientation and distribution of these lineaments were further visualized using rose diagrams generated in Rockwork (version 2018). Lineament density (expressed as frequency per km²) was calculated using ArcGIS version 10.8, providing spatial insights into areas of higher structural permeability.

#### Topographic modelling

A Shuttle Radar Topography Mission (SRTM) Digital Elevation Model (DEM), obtained from the USGS repository at 30-meter resolution, served as the basis for quantifying surface elevation, computing slope gradients, and segmenting watershed and drainage networks. These hydrological layers were derived through ArcGIS 10.8, enhancing the understanding of runoff behavior and potential recharge sites.

#### Vegetation and surface moisture assessment

Sentinel-2 multispectral imagery was utilized to calculate the Normalized Difference Vegetation Index (NDVI) and a moisture index for the study area. Preprocessing steps, including georeferencing and radiometric correction, were performed in the Sentinel Application Platform (SNAP) to ensure high-quality analysis.

#### Land use and land cover mapping

High-resolution (10 m) land use/land cover classification was conducted using the Esri Global Land Cover Map, which leverages processed Sentinel-2 data. This allowed for precise discrimination of crop lands, urban areas, bare soil, and water bodies, informing assessments of surface hydrology and recharge conditions.

All remote sensing datasets were coordinated to the Universal Transverse Mercator (UTM) Zone 35 North projection using the WGS-84 datum, ensuring rigorous geospatial consistency throughout the analysis. Collectively, this integrated workflow of radar, multispectral, and DEM data provided a robust basis for mapping the hydrogeological and structural conditions crucial for groundwater prospecting in Bahariya Oasis.

### Aeromagnetic data

The reduced-to-pole (RTP) aeromagnetic data used in this study were digitized from the 1:500,000 scale aeromagnetic map produced by the Egyptian General Petroleum Company, with a 25 nT contour interval^33^. To ensure high spatial fidelity and mitigate uncertainties associated with digitization and interpolation of contoured aerogeophysical data, high-resolution georeferencing was applied, multiple gridding algorithms were tested, and grid resolution was constrained by contour spacing. Several iterations were applied to the interactive spectral filtering in OASIS Montaj and compared with the original RTP map to ensure high accuracy and fidelity. The primary objectives of analyzing the aeromagnetic map include estimating the basement depth, identifying structural features within the basement rocks, and detecting subsurface faults, all of which contribute to a comprehensive understanding of the area’s hydrogeological characteristics. To isolate local magnetic anomalies, the International Geomagnetic Reference Field (IGRF) values were subtracted to remove regional magnetic influences. Additionally, the magnetic dataset was processed using the Fast Fourier Transform (FFT) technique to perform the reduction-to-the-pole transformation^34^. This transformation helps eliminate distortions in the position, shape, and dimensions of the magnetic anomalies, providing more accurate geological interpretations.

Two primary procedures for magnetic data analysis were implemented to aid groundwater investigations. The first procedure involved structural analysis using two advanced edge-detection methods (TAHG^35^ and MGTHG^36^). After applying the reduced-to-pole (RTP) transformation, upward continuation was used to attenuate short-wavelength magnetic anomalies, following the approach outlined by^37^. This filtering method helps reduce high-frequency noise and emphasizes broader, regional magnetic features^37^. The generated maps reveal the spatial distribution of magnetic susceptibility sources at different depths, allowing for the identification of geologically relevant anomalies. The key aim of this technique is to analyze how the depth of magnetic sources varies across the study area. Each level of upward continuation corresponds to a specific depth range, offering a layered understanding of subsurface magnetic structures. In this research, upward continuation was performed at depths of 1, 2, 3, 4, and 5 km (Fig. 6), highlighting magnetic sources situated below roughly 0.5, 1, 1.5, 2, and 2.5 km, respectively^38,39^. These findings provided valuable insights into deeper magnetic anomalies and enhanced the structural interpretation of the subsurface^39–47^. This approach is essential in groundwater studies for determining the main structural orientations and identifying fault locations, which influence the direction of subsurface water flow. Similar methodologies have been previously employed in groundwater research by various authors^10,19,40^.

**Fig. 2:**
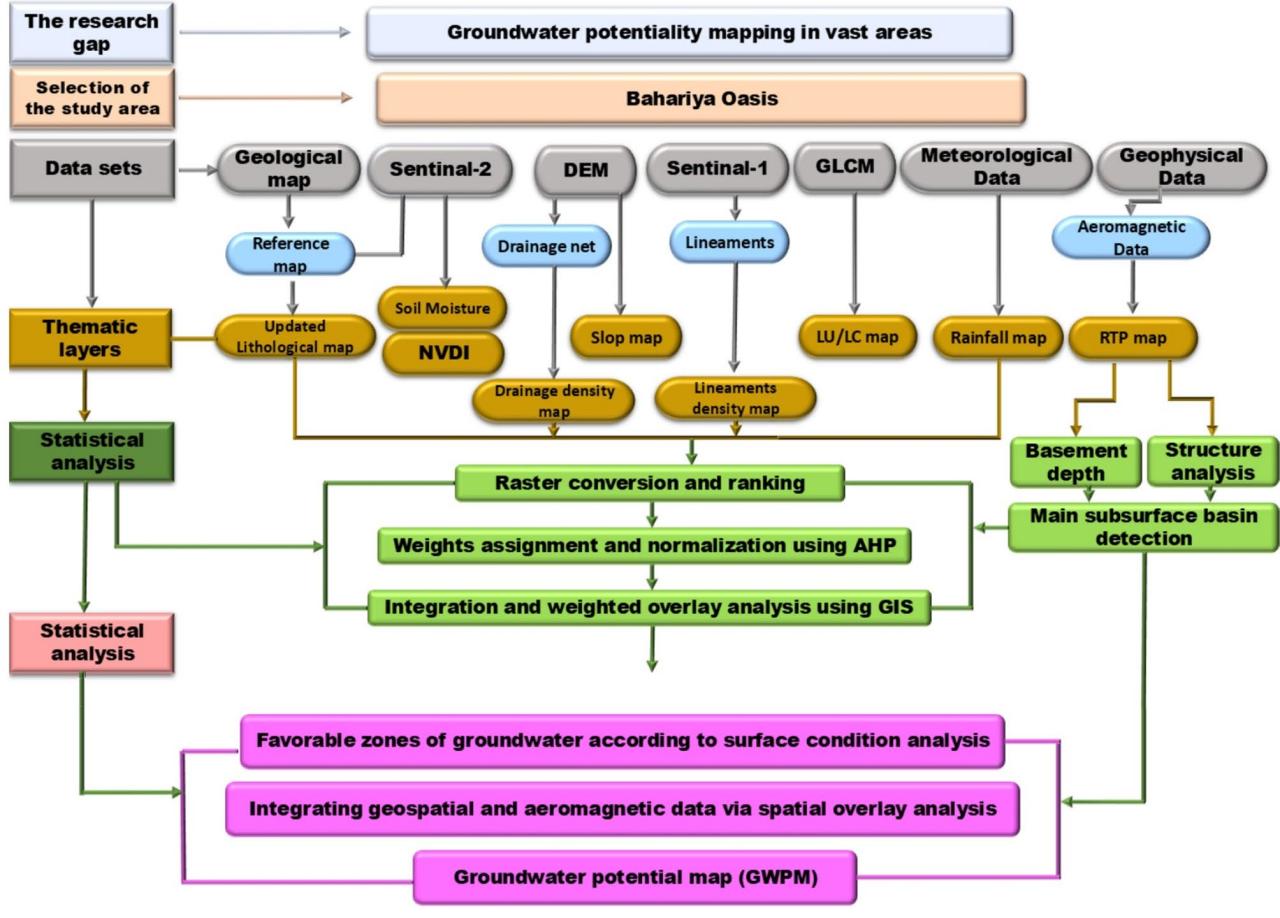
Flowchart of the proposed methodology used in this study.

The Center for Exploration Targeting–Grid Analysis (CET) technique was applied to enhance magnetic image textures and identify structurally complex zones relevant to groundwater exploration. The Structural Complexity (SC) approach evaluates spatial variability and texture attributes to extract linear features corresponding to faults, contacts, and boundaries. Through statistical analysis, phase symmetry detection, and vectorization, CET-derived lineaments provide a robust representation of the structural framework, supporting the identification of zones that may influence groundwater flow and accumulation^48–50^. The second procedure focused on estimating basement depth using the Euler deconvolution method (EUD)^51–53^ and two-dimensional magnetic modeling^54–59^. In this study, Euler deconvolution was applied to the (RTP) magnetic data using a structural index of zero (SI = 0), appropriate for contact-type sources such as faults and lithological boundaries, to delineate structural features potentially controlling groundwater flow and to estimate the depths of their associated magnetic sources. The most coherent clustering of Euler solutions was obtained with a 10-point window and a tolerance of 20^51,52^. This step aimed to define the base of the Nubian aquifer and highlight regions with extensive sedimentary thickness and substantial groundwater reserves.

The datasets used in this study were acquired over different periods. For more information on considerations for data temporal compatibility and data processing steps (see Supplementary). The proposed methodology used in this study is shown in Fig. 2.

**Fig. 3:**
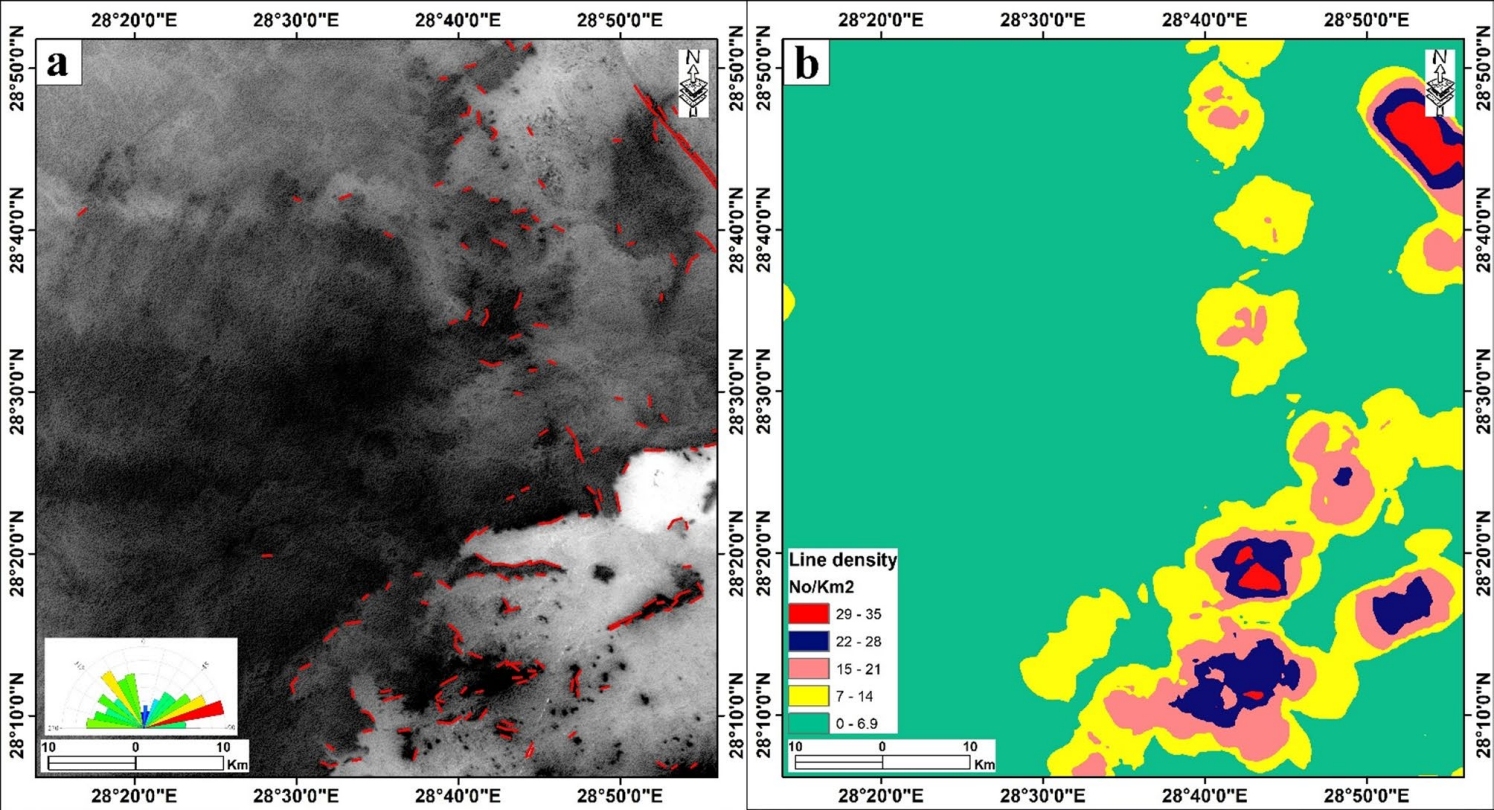
(**a**) Automatic lineaments extraction and rose diagram based on DEM. (**b**) Line density map of the Bahariya area. The figure was created by ArcGIS Desktop 10.8. software; https://www.esri.com/en-us/arcgis/products/arcgis-desktop/overview, ENVI v. 5.3. software; https://www.l3harrisgeospatial.com/Software-Technology/ENVI, Geomatica PCI software and RockWorks v. 18 software; https://www.rockware.com/product/rockworks/.

## Results

This study examines the relationship between surface geological features and drainage systems in the Bahariya region using remote sensing data and subsequently investigates the underlying subsurface structures through aeromagnetic analysis.

### Surface lineaments extraction

Lineaments are mappable linear surface features that reflect subsurface geological structures, including faults, fractures, and lithological boundaries^9,60,61^. Mapping these features is fundamental to geological exploration in the Bahariya area, as they often control the localization of ore deposits and the recharge and movement of groundwater^10^.

In this study, automatic lineament extraction was performed using Sentinel-1B (C-band SAR) imagery (Fig. 3a). The extraction process was governed by specific parameters (Table 1) to optimize feature detection while minimizing noise: the Filter Radius (RADI) determined the sensitivity of the edge detection filter; the Edge Gradient Threshold (GTHR) established the minimum brightness contrast required to define an edge; the Length Threshold (LTHR) filtered out short, insignificant curvilinear features; and the Fitting Error Threshold (FTHR) controlled the precision of curve generation. A rose diagram was subsequently created using Rockworks 18 software to analyze the statistical orientation of the extracted features. Following extraction, the lineament vectors were imported into ArcGIS 10.8 to generate a lineament density map (Fig. 3b), which identified zones of high structural intensity. Accordingly, the predominant surface lineament directions are NE-SW and NW-SE, with minor E-W trending (Fig. 3a). Three-stage post-processing workflow employed: (1) Manual Screening, where high-resolution satellite imagery (Google Earth) was used to identify and delete cultural features such as desert roads and agricultural boundaries; (2) Length Filtering, using a threshold to remove short, disconnected “noise” segments; and (3) Orientation Analysis, where duplicate lineaments were consolidated using a GIS-based proximity analysis. These steps ensure the final lineament density map accurately reflects brittle deformation in the bedrock. This careful post-processing ensured that the final lineament density map (Fig. 3b) primarily reflects geologically significant structural features, thereby increasing its reliability as a proxy for subsurface fracture permeability in the groundwater potential model.

**Fig. 4:**
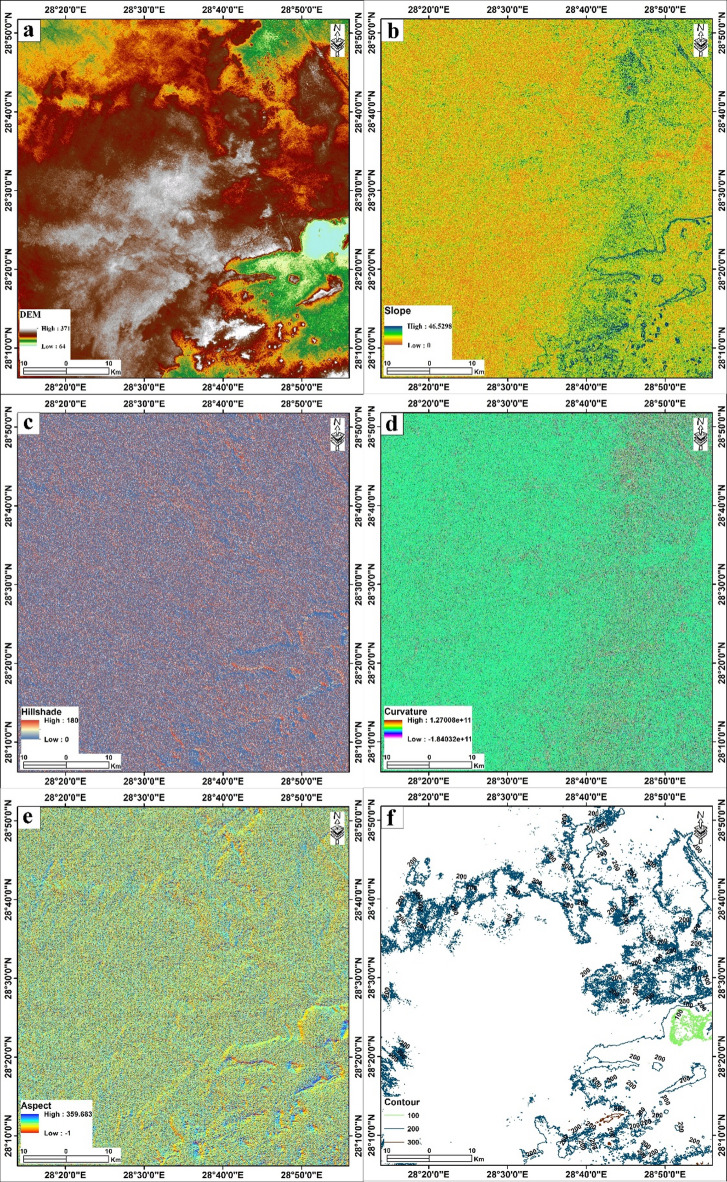
Surface analysis of the Bahariya area (**a**) Elevation of the study area. (**b**) Slope. (**c**) Aspect (**d**) Curvature (**e**) Hillshade (**f**) Contour lines. The figure was created by ArcGIS Desktop 10.8. (https://www.esri.com/enus/arcgis/products/arcgis-desktop/overview/).

**Table 1:** Parameters used in the lineament extraction algorithm.

Data	(RADI)	(GTHR)	(LTHR)	(FTHR)	(ATHR)	(DTHR)
Sentinel-1	20	100	30	3	15	20

The NE–SW trending lineaments, which dominate the Bahariya area, correspond to the major fold axes and fault systems of the Syrian Arc tectonic belt^27^. The NW-SE faults are the second common trend in the Bahariya area. These results are consistent with previous work in the area^28^. Spatially, the highest lineament density is recorded in the Bahariya Fm and the escarpments surrounding the depression (Fig. 1a), where brittle deformation is well preserved. In contrast, the Quaternary alluvium and aeolian sand deposits on the depression floor exhibit a lower density of detectable lineaments due to the masking effect of recent sedimentation (Fig. 3b).

### Digital elevation model and morphometric analysis

The Digital Elevation Model (DEM) serves as the fundamental dataset for analyzing the surface morphology and topographic characteristics of the Bahariya area. In this study, an ASTER DEM was utilized to derive key morphometric parameters, including elevation, aspect, curvature, hillshade, contours, and slope (Fig. 4). These morphometric parameters are not merely descriptive; they actively control the surface water processes, groundwater recharge potential, and land-use suitability in this hyper-arid depression^10,11,62^.

**Fig. 5:**
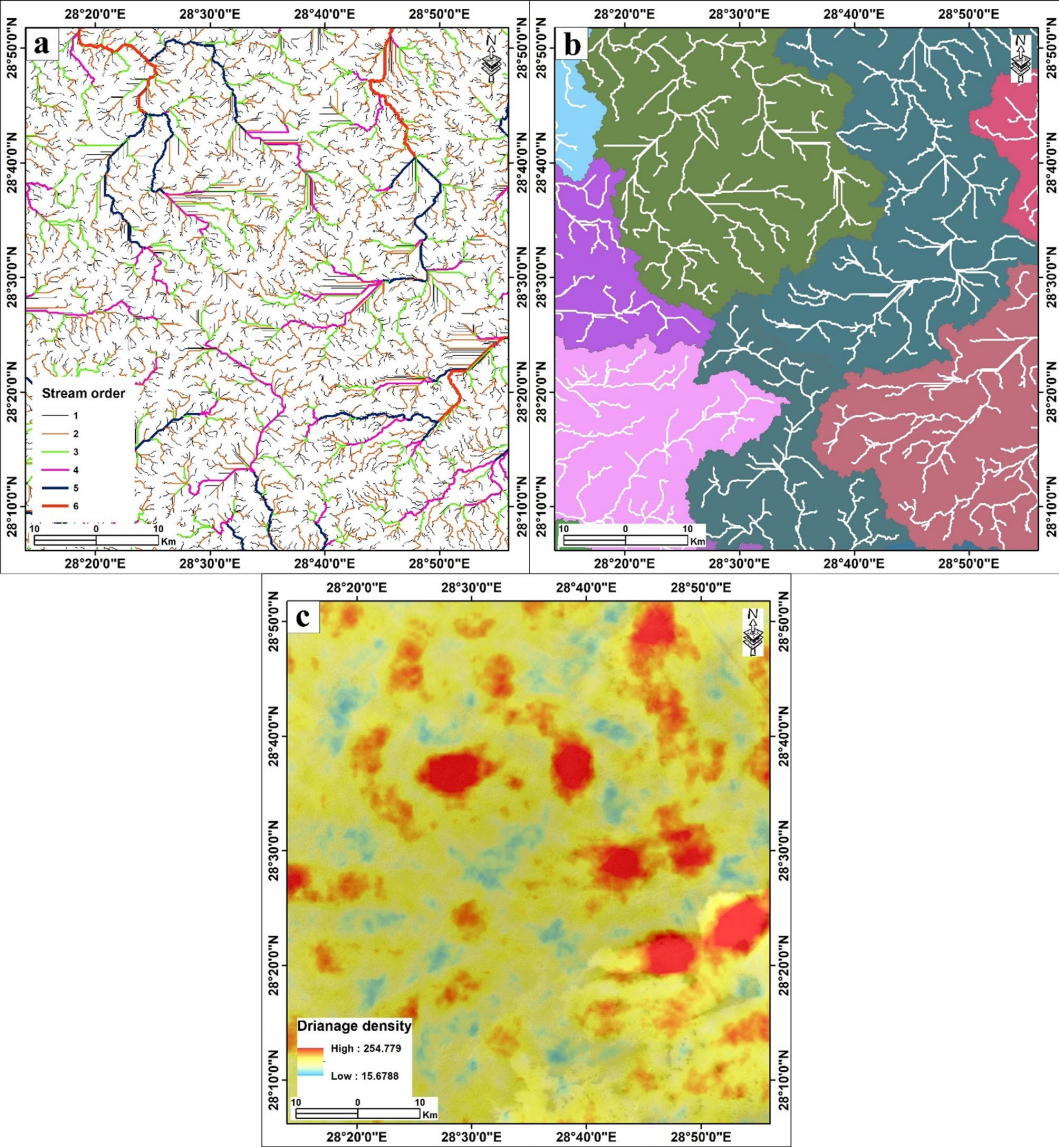
(**a**) Stream order. (**b**) Watersheds and drainage channels. (**c**) Drainage density. The figure was created by ArcGIS Desktop 10.8. (https://www.esri.com/enus/arcgis/products/arcgis-desktop/overview/).

#### Elevation and slope

The DEM analysis reveals that the elevation within the study area ranges from approximately 64 to 371 m above sea level, quantitatively defines the Bahariya Oasis as a closed topographic depression (Fig. 4a). The variation in elevation clearly delineates the geomorphological architecture of the oasis: the lower values (green hues, ~ 64–150 m) correspond to the central depression floor where agricultural activities are concentrated, while the higher values (brown to white hues, > 200 m) represent the surrounding Eocene limestone plateaus and prominent escarpments that enclose the depression. This natural focusing mechanism enhances local groundwater recharge potential, particularly at the foot of escarpments, where runoff velocity decreases, and infiltration capacity is often higher due to the accumulation of coarser sediments.

Slope significantly influences groundwater recharge and the direction of runoff. Accordingly, flatter areas with lower slopes tend to absorb surface water over longer periods, while steeper or moderate slopes generate more runoff^10,11^. The slope gradient analysis indicates values ranging from 0 to 46.5 degrees (Fig. 4b). The spatial distribution of slope confirms the topographic dichotomy of the area: the depression floor is characterized by gentle, nearly flat terrain (0–5°, blue zones), suitable for agriculture and habitation, while the bounding escarpments exhibit steep gradients (> 20°, yellow to red zones), which control runoff velocity and flash flood routing into the basin. The slope analysis (Fig. 4b) complements the elevation data by identifying zones of transport versus accumulation. The steep slopes (> 20°) along the escarpments facilitate rapid runoff, preventing significant infiltration on the basin margins. Conversely, the gentle gradients (0–5°) of the depression floor promote water stagnation, allowing for potential infiltration or, in discharge zones, evaporation. This relationship is critical for understanding the “Dual Role” of the depression floor: it is both a potential recharge zone during rare flood events and a discharge zone where the shallow water table is vulnerable to evapotranspiration, leading to soil salinization.

#### Hillshade and curvature

The hillshade and curvature (Fig. 4c, d) maps serve as proxies for structural and erosional interpretation. The linear features enhanced in the hillshade map often correlate with the regional fault systems (NE-SW and NW-SE trends) as discussed in the lineament analysis (Supplementary Fig. 1; Fig. 3). These structural weaknesses likely guided the erosional processes that excavated the depression. Furthermore, the curvature map highlights drainage lines (concave zones) that act as preferential pathways for surface flow. These pathways often overlie structural lineaments, suggesting that surface drainage may be structurally controlled, potentially linking surface runoff to deeper fracture networks in the underlying Nubian Sandstone Aquifer.

The curvature represents the second derivative of the surface elevation, highlighting the rate of change in slope (Fig. 4d). The map distinguishes between convex profiles (positive values) and concave profiles (negative values). This distinction is vital for hydrological modeling, as concave surfaces typically function as accumulation zones for runoff and sediment, whereas convex surfaces facilitate dispersal and erosion. To visualize the relief and structural texture of the terrain, a hillshade map was generated using a single illumination source positioned at an azimuth of 315° and an altitude of 45°. The resulting map, with values ranging from 0 to 180, enhances the visibility of linear features, escarpment edges, and drainage incisions by simulating surface shadows, thereby aiding in the interpretation of structural lineaments (Fig. 4e).

#### Aspect and contour

The aspect map, which identifies the downslope direction of the maximum rate of change in value from each cell to its neighbors, shows values ranging from − 1 to 359.7 degrees (Fig. 4e). This parameter is critical for understanding the direction of surface runoff and the distribution of solar insolation, which influences local evaporation rates. Moreover, Topographic contours were extracted from the DEM to provide a vector-based representation of elevation changes (Fig. 4f). The map illustrates the steep gradients associated with the depression margins, particularly delineated by the 200-meter contour line, which often marks the transition from the depression floor to the escarpment face. From an applied perspective, the aspect (Fig. 4e) and contour (Fig. 4f) maps offer guidance for agricultural and urban planning. The dominance of specific slope facings (aspect) influences microclimatic conditions; for instance, south-facing slopes experience higher solar radiation and evaporation, which can potentially increase crop water requirements. Meanwhile, the contour and slope data delineate the physical limits of agricultural expansion. The flat, low-elevation zones are already heavily utilized, suggesting that future development must carefully balance expansion into slightly higher, distinctive terrain with the increased cost of water lifting and the risk of encroaching on flash-flood pathways.

Finally, the morphometric analysis confirms that Bahariya’s potential as a groundwater-dependent ecosystem is closely tied to its topography. The depression functions as a natural basin for sediments and water, with its edges facilitating rapid runoff and its center serving as the primary zone for accumulation, recharge, and discharge.

### Groundwater recharge and hydrological analysis based on DEM

This study utilized Digital Elevation Model (DEM) data to model the hydrological framework of the Bahariya area, enabling the extraction of critical drainage parameters, including stream networks, watershed basins, and drainage density (Fig. 5). Based on SRTM DEM data, drainage networks were created using the ArcGIS Hydro tool^9–11,63^.

**Fig. 6:**
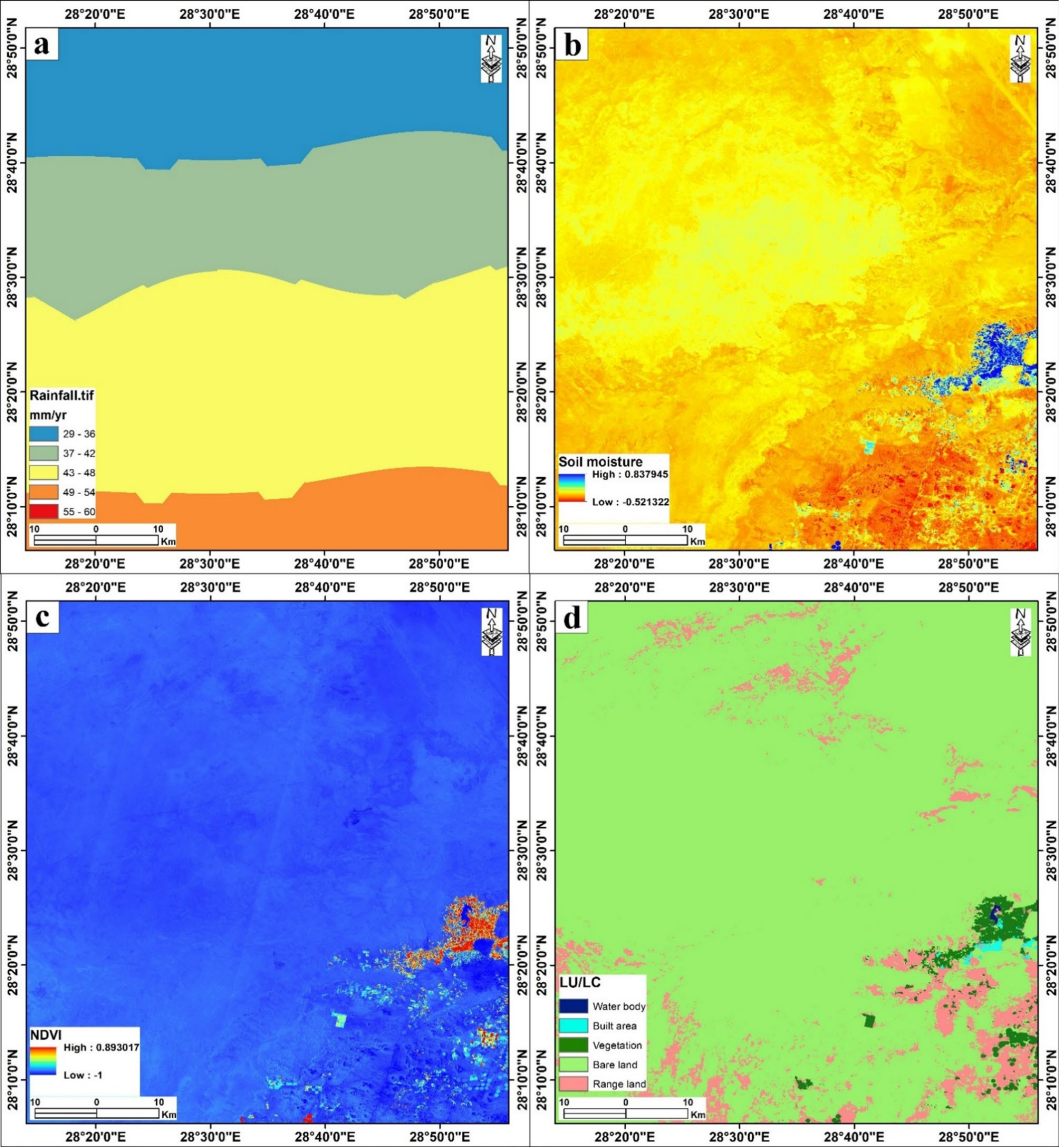
(**a**) Rainfall. (**b**) Soil moisture. (**c**) NDVI. (**d**) LU/LC of the study area. The figure was created by ArcGIS Desktop 10.8. (https://www.esri.com/enus/arcgis/products/arcgis-desktop/overview/).

#### Stream network and order

The drainage network was generated using the ArcGIS Hydro toolbox. The ‘Fill’ tool was first applied to remove spurious sinks (Supplementary Fig. 2a). Flow direction and accumulation were then calculated (Supplementary Fig. 2b, c). A constant threshold of 500 contributing cells was applied to the flow accumulation raster to define the stream network, a value determined to best represent the perennial and ephemeral channel system visible in satellite imagery. The analysis reveals a well-developed dendritic drainage pattern, characteristic of the erosional landscape on the Eocene limestone plateaus (Fig. 5a). The stream order classification (Strahler method) identified streams ranging from 1 st to 6th order (Fig. 5a; Table 2). The higher-order streams (4th, 5th, and 6th orders), depicted in thick violet, blue, and red lines, respectively (Fig. 5a), represent the main wadi channels that collect runoff from the plateau and funnel it into the depression. Unlike the Nile Valley systems^10^, these channels do not drain to the river but terminate within the closed basin of the oasis, playing a vital role in directing occasional flash flood water towards potential recharge zones on the depression floor.

**Table 2:** Statistics of stream length from the Bahariya area, Western Desert, Egypt.

Stream order	Count streams	Minimum (m)	Maximum (m)	Average (m)	Sum (km)	St. deviation (m)
1	7326	27.213	9152.647	998.218	7312.944	919.588
2	3498	13.571	7378.417	958.182	3351.271	878.884
3	1768	13.631	8462.312	903.33	1597.088	902.207
4	874	27.283	6750.73	770.533	673.447	742.695
5	663	27.1	5909.882	813.066	539.063	731.303
6	259	41.004	5195.984	742.806	192.387	714.857

#### Watershed basins

The study area was partitioned into distinct watershed basins based on flow direction and accumulation points. Figure 5b illustrates these sub-basins in different colors, effectively mapping the catchment areas that feed into the oasis. The boundaries of these basins coincide with the high structural ridges and escarpments, defining the limits of the hydrological system. Understanding these catchments is crucial for calculating total runoff volumes during storm events and for siting flood protection structures.

#### Drainage density

Drainage density, defined as the total length of streams per unit area, is a key indicator of surface permeability and runoff potential^9–11^. Figure 5c presents the drainage density map, with values ranging from low (yellow-blue zones) to high (red zones). High drainage density zones were observed on the steep escarpments and parts of the limestone plateau (Fig. 5c). These areas are characterized by impermeable rocks and steep slopes, which promote rapid surface runoff rather than infiltration. Whereas the low drainage density zones were found primarily on the depression floor. These zones are associated with permeable Quaternary sediments (aeolian sands and alluvial deposits) and gentle gradients, which facilitate the infiltration of surface water, thereby enhancing direct groundwater recharge to the shallow aquifer.

In summary, the integration of slope (Fig. 4b) and drainage network analysis (Fig. 5) highlights the dual hydrological nature of the Bahariya depression. The high-density drainage network on the plateau acts as a collection system, rapidly transporting rainwater to the basin margins. Upon reaching the low-density, low-slope areas of the depression floor, flow velocity decreases, allowing for the percolation of accumulated runoff. This process suggests that the edges of the depression, where major wadis debouch onto the sandy plains, represent the most promising zones for structural measures to enhance artificial recharge and mitigate flash flood hazards.

### Soil moisture, rainfall, NDVI, and LU/LC factors

The integration of soil moisture, rainfall, vegetation indices (NDVI), and land use/land cover (LU/LC) forms the core of the hydrogeological assessment for the Bahariya area (Fig. 6). These variables were prioritized based on their direct influence on groundwater recharge and accumulation potential.

#### Rainfall distribution

Rainfall is the primary renewable source for aquifer replenishment, though it is sparse in this hyper-arid region. The spatial rainfall map (Fig. 6a) shows a clear latitudinal gradient, with annual precipitation increasing from north (29–36 mm/yr, blue) to south (46–53 mm/yr, red). While the overall magnitude is low compared to coastal regions, the southern part of the study area receives relatively higher rainfall, suggesting a marginally higher potential for direct recharge in the southern sub-basins compared to the northern sectors.

#### Soil moisture (SM)

Soil moisture is considered the second most critical indicator of near-surface hydrological conditions in arid environments^9,10^. A simple normalized difference moisture index (NDMI) was calculated using Band 8 A (NIR) and Band 11 (SWIR) as (B8A - B11)/(B8A + B11) to estimate relative surface moisture content. The derived soil moisture map (Fig. 6b) reveals a distinct spatial pattern: high moisture values (reaching 0.81, shown in blue) are concentrated within the depression floor, particularly in agricultural zones. In contrast, the surrounding plateaus and escarpments exhibit very low moisture content (yellow-orange hues, dropping to −0.57). The elevated moisture in the depression is a direct proxy for shallow groundwater tables, irrigation return flows, and potential capillary rise, making it a primary indicator for groundwater occurrence.

#### Normalized difference vegetation index (NDVI)

The NDVI serves as a robust bio-indicator for groundwater availability, especially where vegetation relies on phreatic water. The NDVI was calculated using Sentinel-2 Bands 8 (NIR) and 4 (Red) using the standard formula: NDVI = (B8 - B4)/(B8 + B4). The calculated NDVI map (Fig. 6c) ranges from − 1.0 to 0.89. High NDVI values (green/red clusters) are strictly confined to the agricultural plots and natural oases within the depression floor, directly overlying the shallow aquifer. The vast majority of the study area (plateau and desert plains) exhibits low to negative NDVI values (blue), confirming the absence of vegetation and surface moisture. The strong correlation between high NDVI and the depression floor further supports the delineation of the primary groundwater discharge and utilization zone.

#### Land use/Land cover (LU/LC)

The LU/LC classification (Fig. 6d) provides a functional segmentation of the landscape. The analysis indicates that “Bare Land” (yellow) dominates the region, covering the plateaus and desert fringes. “Crops” (blue) and “Built-up Areas” (magenta) are clustered exclusively within the depression, aligning perfectly with the anomalies in soil moisture and NDVI. This distribution highlights that anthropogenic activity and vegetation are entirely dependent on the groundwater resources of the depression, confirming the closed-basin hydrological cycle of the oasis.

Synthesis.

From the aforementioned results, these thematic layers illustrate a convergent hydrological system: the depression acts as the focal point where rainfall (though limited), soil moisture, and vegetation intersect. The surrounding “bare” plateaus serve as the catchment, while the depression floor serves as the storage and discharge zone, validating its classification as the area with the highest groundwater potential.

### Aeromagnetic results

Interpreting geophysical datasets plays a central role in producing reliable groundwater potential maps (GWPMs). By employing an integrated and comprehensive workflow, this study enhances the accuracy of groundwater assessments and strengthens the overall delineation of groundwater potential zones. Aeromagnetic data were first employed to map basement configurations and delineate subsurface fault systems. Establishing these structural frameworks is essential for understanding the hydrogeological setting of the study area and for assessing how structural controls influence groundwater occurrence and movement.

**Fig. 7:**
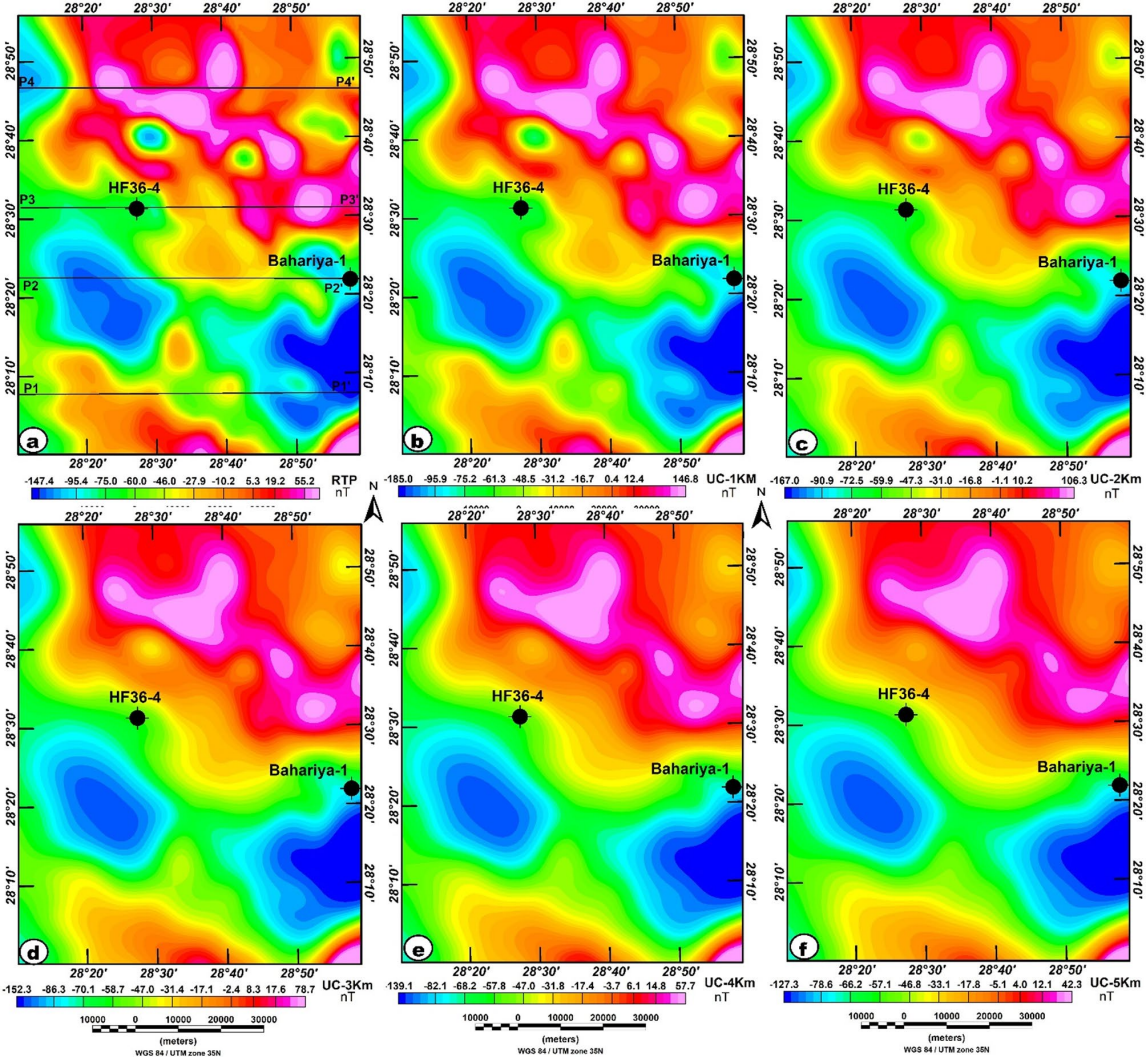
(**a**) RTP map; (**b**) UC-RTP at 1 km; (**c**) UC-RTP at 2 km; (**d**) UC-RTP at 3 km; (**e**) UC-RTP at 4 km; and (**f**) UC-RTP at 5 km of the study area, highlight magnetic sources with depth and enhance the structural interpretation of the subsurface. The figure was generated using Geosoft Oasis montaj (v8.3.3) software (Seequent; https://www.seequent.com/products-solutions/oasis-montaj/).

The RTP map illustrates a complex distribution of magnetic anomalies across the Bahariya area, with values ranging from approximately − 147 nT to + 55 nT (Fig. 7a). This color-scale distribution reflects variations in basement depth, lithology, and potential intrusive bodies. Large, continuous magnetic lows appear prominently in the southern, southwestern, and southeastern regions of the map, particularly near the Bahariya-1 well, corresponding to the Bahariya depression. These lows likely correspond to deeply buried basement surfaces. The thick sedimentary cover (low magnetization) overlying these areas is consistent with the view that magnetic lows represent deep basement or thick sedimentary layers^10,19,40^. The broad, smooth nature of these lows suggests regional subsidence zones or structurally downthrown fault blocks.

Strong positive anomalies dominate the central and north-central portions. These areas include the region north of the HF36-4 well. These highs likely represent uplifted basement blocks, shallow magnetic sources, or igneous intrusions. The steep gradients around these highs indicate sharp structural boundaries, likely faults or contacts. However, the RTP map shows several dominant structural trends, including NW–SE trends, evident in the alignment of alternating high and low patches. Suggests major NW–SE fault systems influencing basement geometry.

Additionally, NE–SW Trends, subtle yet present in the central sector, mark possible cross-cutting shear zones. In addition to localized E–W trends, observed where the strike of anomalies changes direction. These intersecting structural systems likely create favorable groundwater traps, especially where downthrown blocks align with fracture corridors. The alternating high-low pattern suggests the presence of multiple structural blocks, forming compartmentalized aquifer zones that govern groundwater movement and accumulation^64^.

The upward-continuation (UC) maps at heights ranging from 1 km to 5 km progressively suppress short-wavelength anomalies and enhance deeper magnetic sources, allowing the large-scale basement architecture to be more clearly identified (Fig. 7b-f). Based on the commonly applied approximation that upward continuation to a height z primarily reflects sources at roughly z/2^38,39^, the estimated equivalent source depths are approximately from 0.5 km to 2.5 km. At lower continuation levels (1–2 km), remnants of shallow features remain slightly visible, but the prominent magnetic high near borehole HF36-4 continues to appear with minimal attenuation, indicating a deeply rooted, high-susceptibility source. As the continuation height increases (3–5 km), the anomaly field becomes smoother and dominated by long-wavelength features. The persistence and lateral expansion of the central magnetic high across all levels strongly suggest the presence of a substantial intrusive body or uplifted basement block.

In contrast, the broad magnetic low surrounding the Bahariya-1 well becomes increasingly coherent with height, delineating a deep structural depression likely filled with thick, low-magnetization sedimentary units. By the highest continuation levels, only these major deep-seated features remain, confirming a distinct contrast between the uplifted magnetic block in the north-central area and the deep sedimentary basin in the south and southeast. This interpretation aligns with regional structural trends and highlights potential hydrogeological zones, particularly where deep basins may serve as favorable groundwater repositories^30^.

However, qualitative interpretation of RTP and upward-continued maps alone can be challenging and may yield ambiguous or non-unique results^36,65^. To enhance the clarity of structural interpretation for groundwater exploration, the TAHG and MGTHG edge-detection filters were applied to the RTP and UC magnetic data. Recent studies have confirmed that these filters are powerful tools for extracting key geological structures from complex magnetic datasets, thereby improving the identification of subsurface features that influence groundwater potential^39,66–68^.

**Fig. 8:**
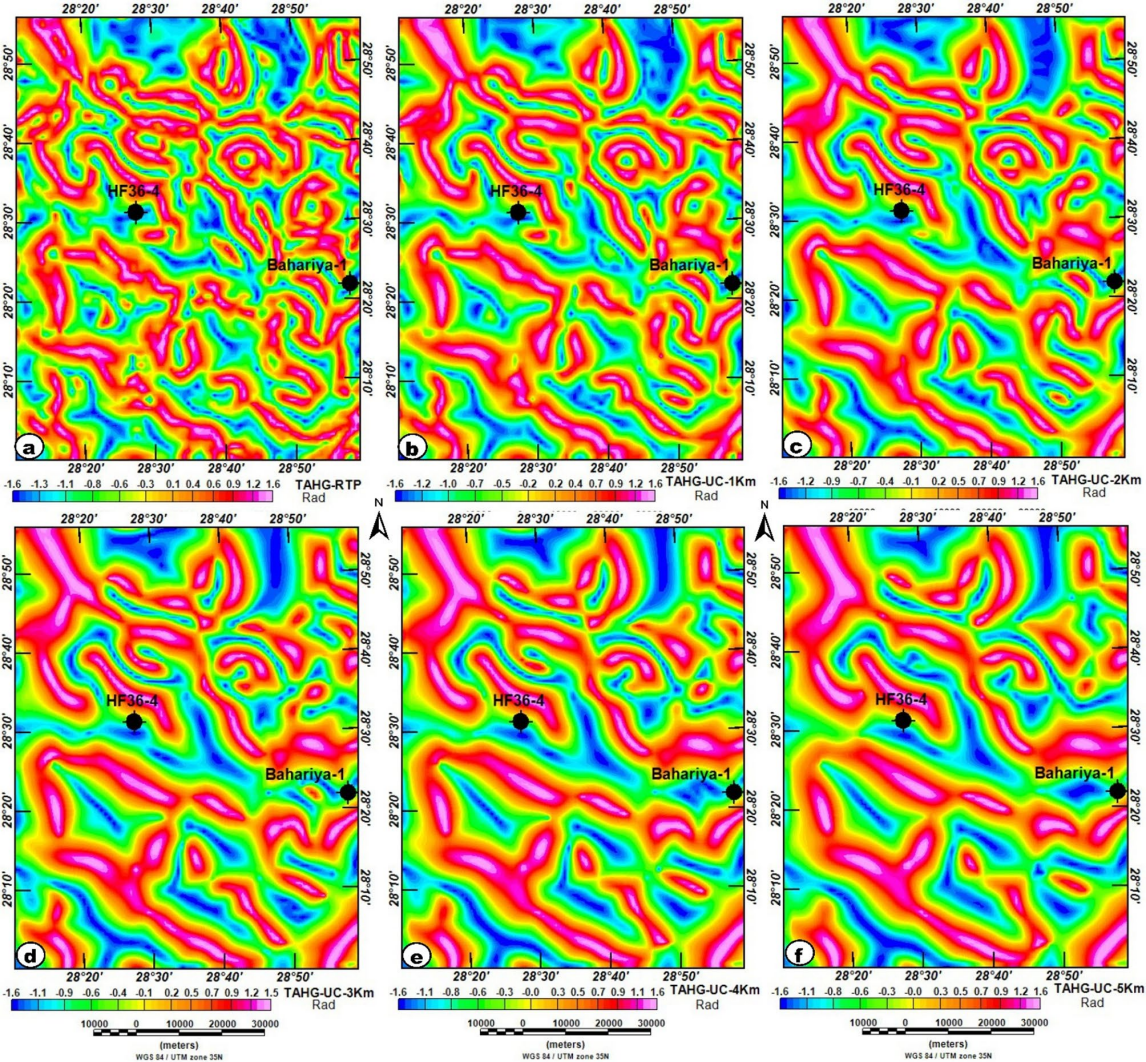
TAHG maps of the study area: (**a**) TAHG-RTP; (**b**) TAHG-UC at 1 km; (**c**) TAHG-UC at 2 km; (**d**) TAHG-UC at 3 km; (**e**) TAHG-UC at 4 km; and (**f**) TAHG-UC at 5 km maps show the boundaries of subsurface source bodies. The figure was generated using Geosoft Oasis montaj (v8.3.3) software (Seequent; https://www.seequent.com/products-solutions/oasis-montaj/).

**Fig. 9:**
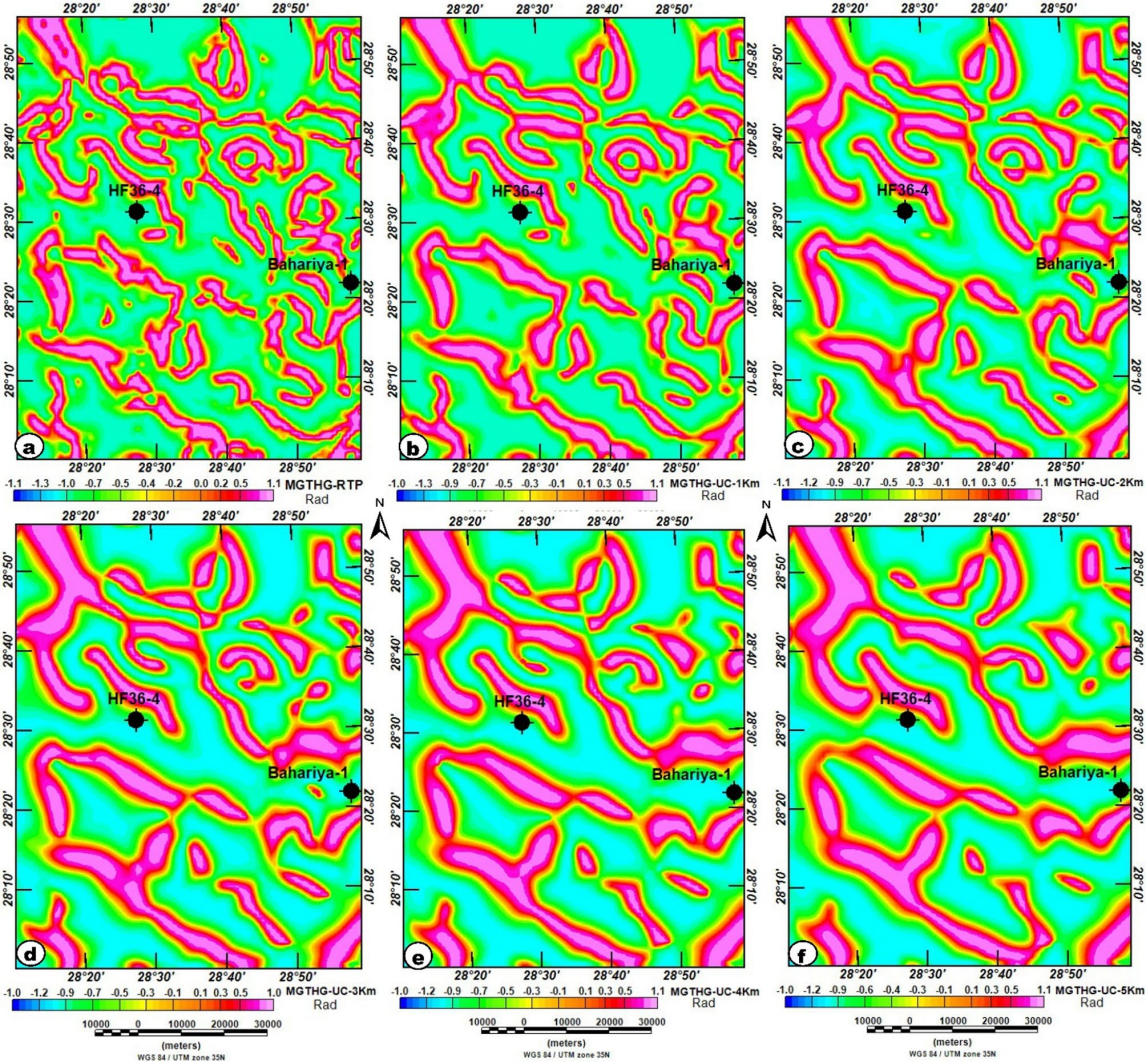
MGTHG maps of the study area: (**a**) MGTHG-RTP; (**b**) MGTHG-UC at 1 km; (**c**) MGTHG-UC at 2 km; (**d**) MGTHG-UC at 3 km; (**e**) MGTHG-UC at 4 km; and (**f**) MGTHG-UC at 5 km maps, show the boundaries of subsurface source bodies. The figure was generated using Geosoft Oasis montaj (v8.3.3) software (Seequent; https://www.seequent.com/products-solutions/oasis-montaj/).

Figures 8 and 9 present the outcomes of applying the TAHG and MGTHG techniques to the RTP dataset. These filtering methods effectively delineate magnetic source boundaries and structural trends throughout the study area. They produce high-resolution, reliable results, contributing significantly to the interpretation of subsurface geological structures. One of the main strengths of these techniques is their ability to enhance peak responses at source edges, thereby improving the clarity of both pronounced and subtle magnetic anomalies^67,69,70^.

**Fig. 10:**
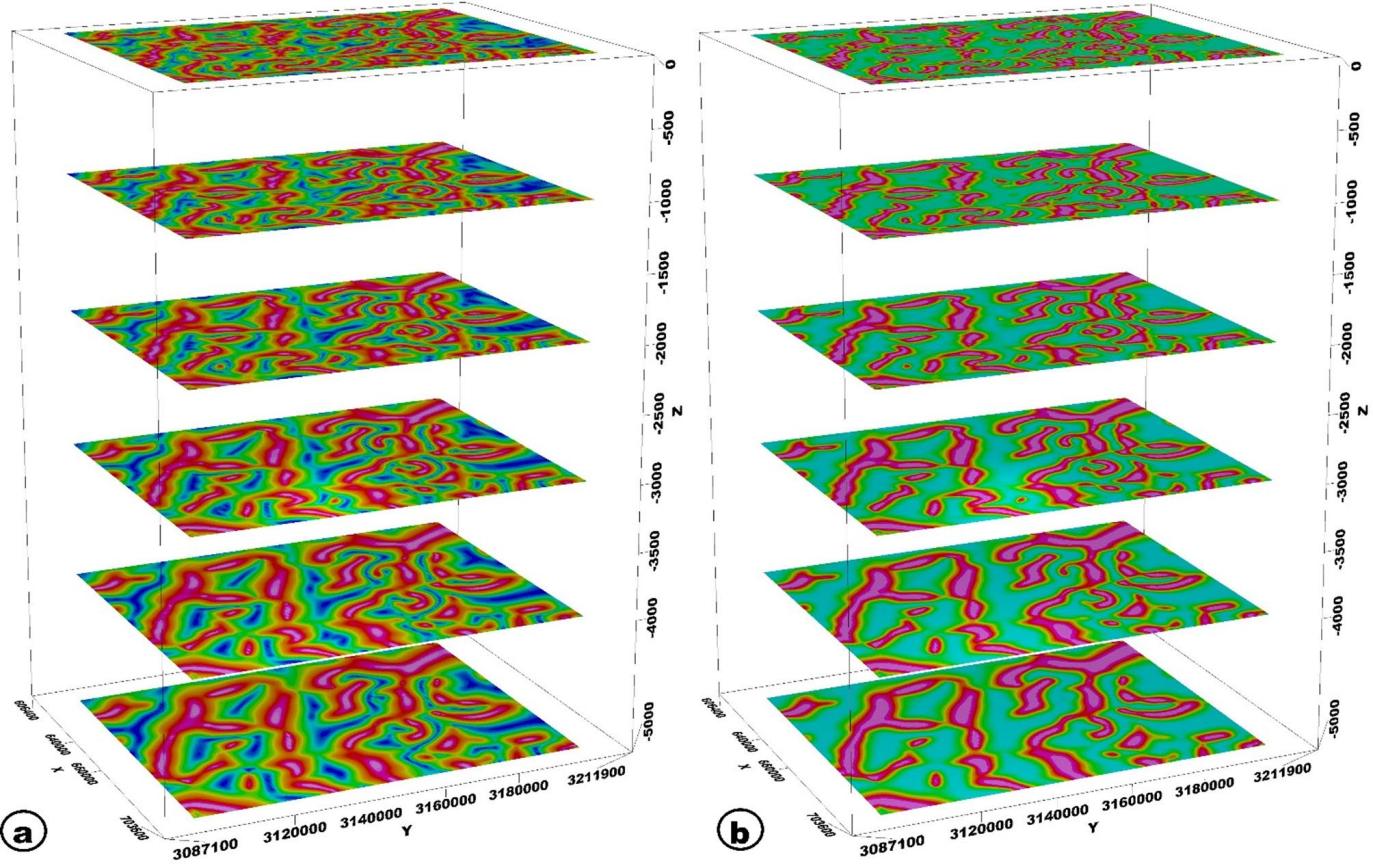
3D plane view of (**A**) TAHG maps, (**B**) MGTHG maps of the study area, showing the change of magnetic anomalies with depth, as the UC level increases. The figure was generated using Geosoft Oasis montaj (v8.3.3) software (Seequent; https://www.seequent.com/products-solutions/oasis-montaj/).

**Fig. 11:**
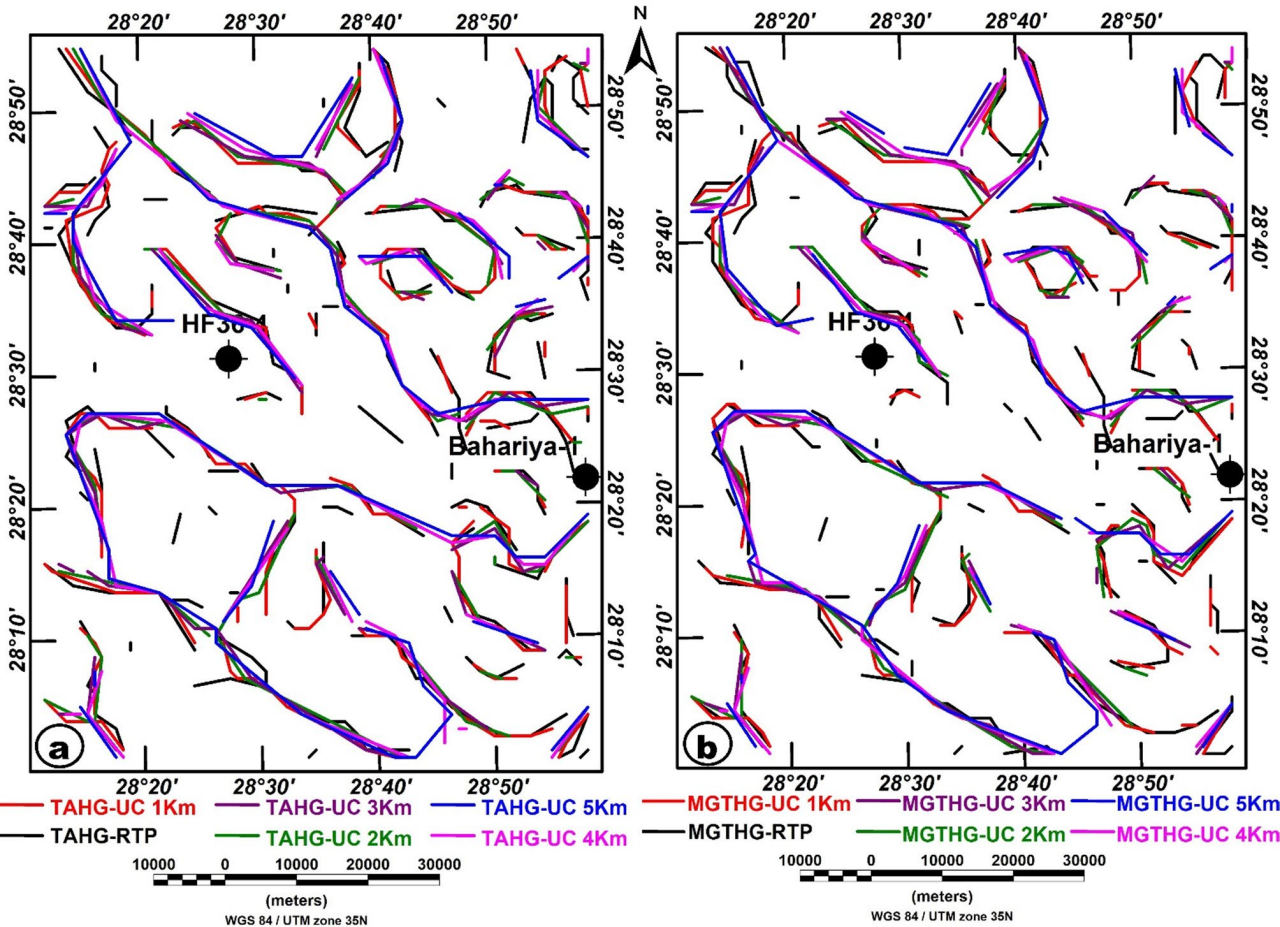
CET structural maps of (**A**) TAHG-RTP; (**B**) TAHG-UC at 1 km; (**C**) TAHG-UC at 2 km; (**D**) TAHG-UC at 3 km; (**E**) TAHG-UC at 4 km; (**F**) TAHG-UC at 5 km of the study area, show the lineaments produced by two advanced edge-detection filters. The figure was generated using Geosoft Oasis montaj (v8.3.3) software (Seequent; https://www.seequent.com/products-solutions/oasis-montaj/).

**Fig. 12:**
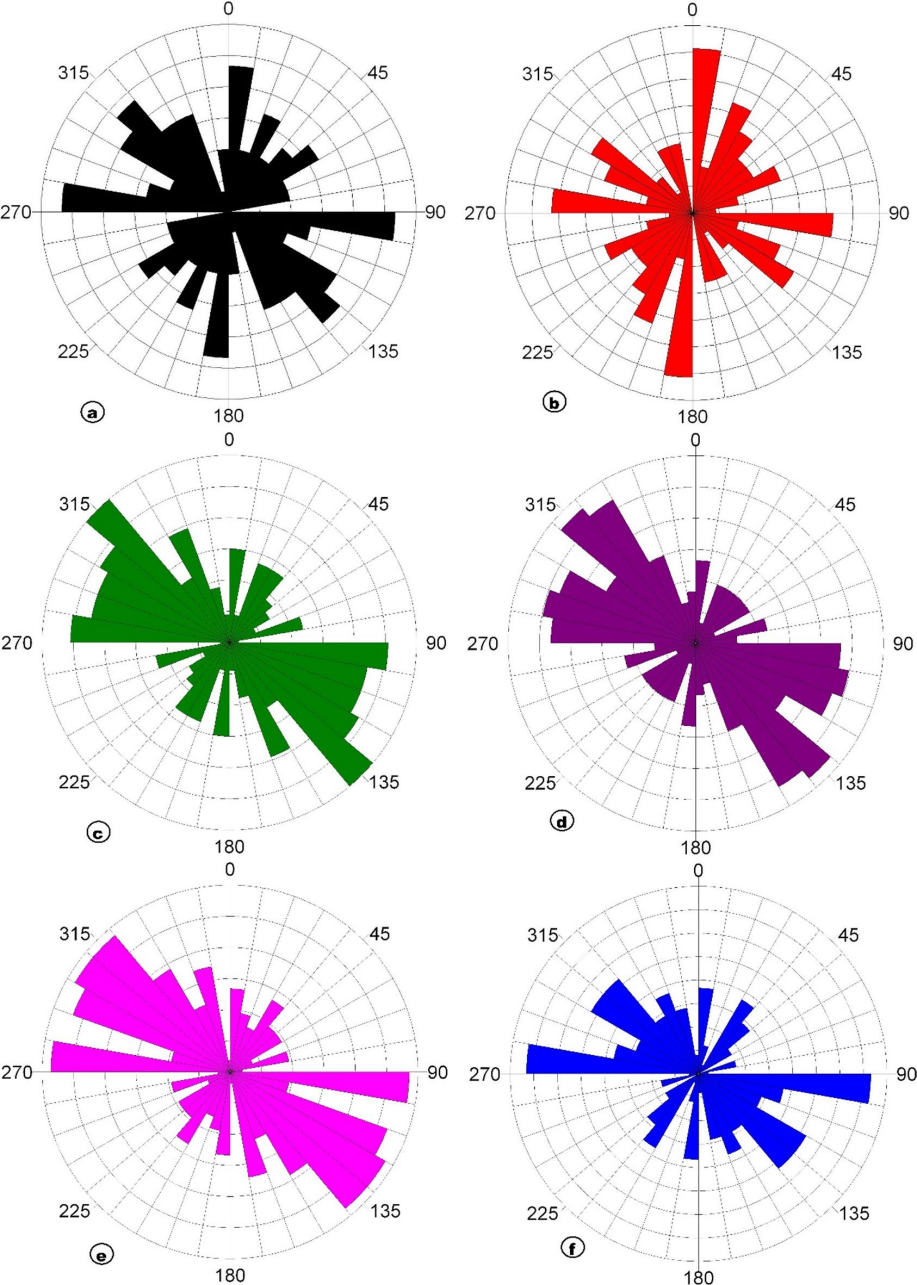
Rose diagrams for the TAHG and MGTHG: (**a**) RTP; (**b**) UC at 1 km altitude; (**c**) UC at 2 km altitude; (**d**) UC at 3 km altitude; (**e**) UC at 4 km altitude; and (**f**) UC at 5 km altitude. The figure was created using RockWare (2017) (https://www.rockware.com/product/rockworks).

The TAHG and MGTHG-RTP and related UC maps at various depths (1–5 km) in Figs. 7 and 8 reveal dominant ENE–WSW, NE–SW, and NW–SE magnetic lineaments. These structural orientations correspond with regional fault systems that may serve as preferential pathways for groundwater flow. The multi-depth application of TAHG and MGTHG filtering offers a pseudo-3D view of the subsurface, enabling differentiation between shallow and deep structures (Fig. 10). Overall, these results highlight the effectiveness of advanced magnetic filtering techniques in mapping hydrogeologically significant features and support their integration into groundwater exploration and well-siting strategies^10^. CET-derived structural maps generated from the TAHG and MGTHG filters applied to the RTP magnetic data and its upward continuations (1–5 km) delineate dense networks of linear features across the study area (Fig. 11). These features display variable continuity and length and are consistently resolved across multiple filtering levels. Rose diagrams constructed from CET-extracted lineaments (Fig. 12) show that the dominant orientations identified in both the TAHG and MGTHG outputs for the RTP and 1-km upward-continued data are ENE–WSW, NE–SW, and NW–SE, with a subordinate N–S orientation. With increasing upward-continuation height (2–5 km), NW–SE–oriented lineaments become increasingly prominent and laterally continuous, while NE-SW-oriented features decrease in relative frequency and clarity. The orientation distributions vary systematically with continuation level, indicating depth-dependent differences in the expression of structural trends.

**Fig. 13:**
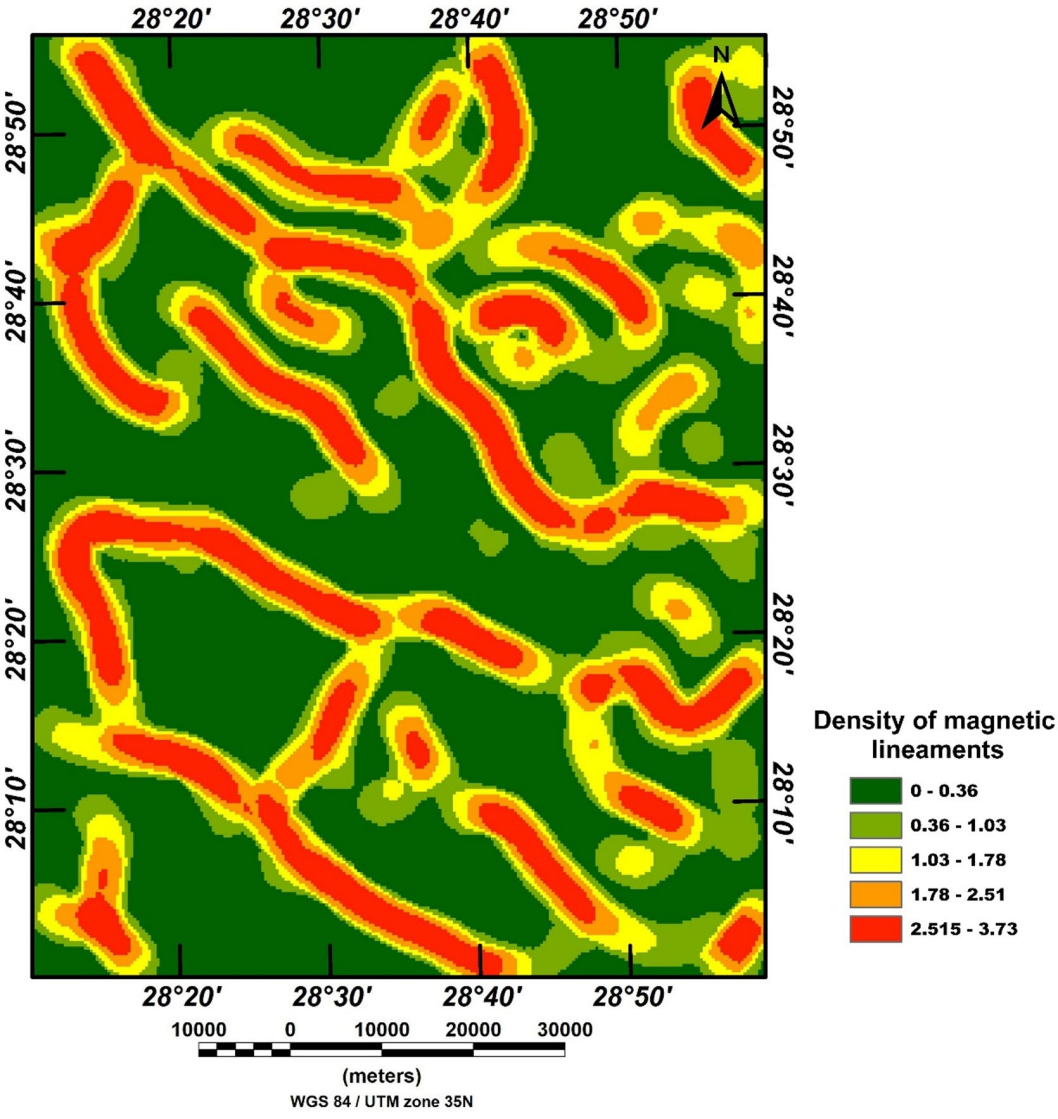
Magnetic Lineament Density map, produced using CET grid analysis. The figure was created by ArcGIS Desktop 10.8. (https://www.esri.com/enus/arcgis/products/arcgis-desktop/overview/).

Figure 13 presents the magnetic lineament density map generated through CET grid analysis, highlighting zones of structural complexity within the study area. High-density regions, shown in red and orange, indicate clusters of closely spaced magnetic lineaments typically associated with faulted and fractured bedrock. These zones enhance secondary porosity and permeability, making them favorable targets for groundwater accumulation and movement.

The dominant structural trends observed are consistent with the regional tectonic fabrics of the Central Eastern Desert and reflect multiple deformation episodes^30,71–81^. The spatial correspondence between high-density lineament zones and these structural trends underscores their potential as conduits for subsurface fluid flow. Conversely, low-density areas (green) likely represent more intact, less permeable bedrock. Overall, this map provides a valuable framework for identifying promising zones for groundwater exploration and guiding future hydrogeological investigations.

**Fig. 14:**
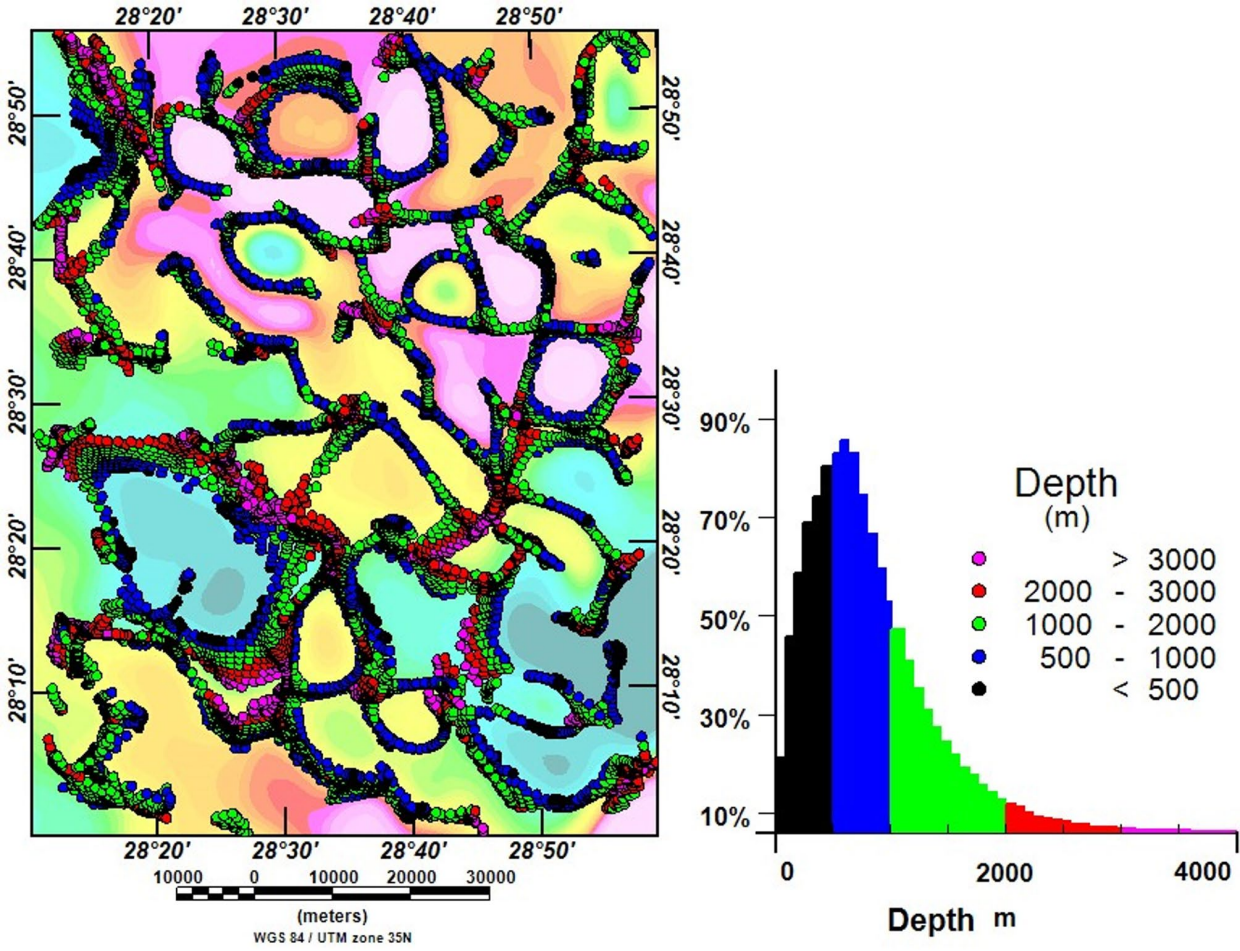
EUD depth solutions of the study area using SI = 0, and the histogram of depth solutions. The figure was generated using Geosoft Oasis montaj (v8.3.3) software (Seequent; https://www.seequent.com/products-solutions/oasis-montaj/).

Figure 14 presents the 3D Euler deconvolution results for the study area. This approach was applied to delineate the spatial distribution of magnetic lineaments and to estimate the depths to the upper boundaries of the causative magnetic sources, which are interpreted here as subsurface structural features. The most coherent clustering of Euler solutions was obtained with a structural index of η = 0, a 10-point window, and a tolerance of 20. The estimated depths range from near-surface values (~ 0 m) to more than 3000 m, with most solutions clustering around approximately 1000 m, indicating that the majority of the mapped lineaments are relatively shallow features. Structurally, the dominant trends are NE–SW to ENE–WSW, forming the main fabric of the area. A pronounced NW–SE set cross-cuts this dominant fabric, locally linking and offsetting NE–SW lineaments and becoming more apparent where deeper solutions (green–red–magenta classes) are concentrated. By contrast, N–S–oriented structures are sparse and occur mainly as minor features.

The 2D magnetic models provide a subsurface picture of the study area by simulating the observed magnetic response along selected profiles. Four 2D magnetic forward models (P1–P1′, P2–P2′, P3–P3′, and P4–P4′) were developed using the GM-SYS modelling module^55^, to provide a geologically representative model of the sedimentary cover and underlying basement architecture. Because the basement complex is not exposed anywhere within the study area and remains entirely subsurface, its composition and structural character are complex to constrain directly; therefore, magnetic modeling is an important tool for improving the geological interpretation. A direct subsurface record on the basement surface is available from only two boreholes in the study area, “HF36-4” and “Bahariya-1”, which indicate basement depths of approximately 2106 m and 1535 m, respectively^77^. Previous geological and geophysical investigations in adjacent areas indicate that the basement is predominantly granitic in composition^75,77,82^. Because the sedimentary cover is essentially non-magnetic, these constraints were incorporated into the modelling to reduce ambiguity and produce models that more realistically represent the subsurface. For the modelling, the basement was assigned an average magnetic susceptibility of about 0.007 cgs^75,77,82^, consistent with the susceptibility range reported for granitic rocks by^83^.

**Fig. 15:**
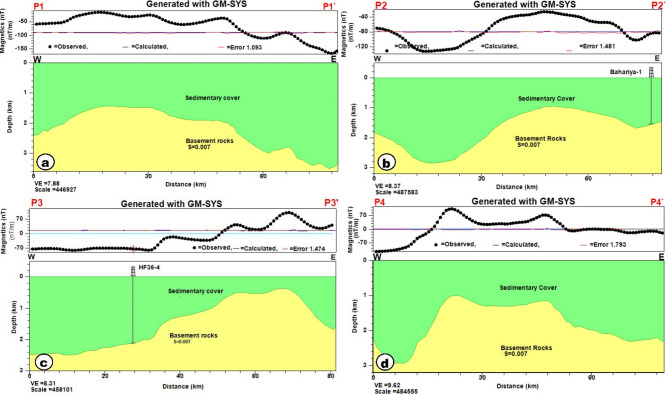
The 2D magnetic susceptibility model along the profile (**A**) P1-P1′, (**B**)P2-P2′, (**C**)P3-P3′,(**D**)P4-P4′. The figure was generated using GM-SYS 2D software (Seequent; https://www.seequent.com/products-solutions/oasis-montaj/).

Figure 15; Table 3 summarize the results of the 2D magnetic modelling. Basement depth ranges from ~ 0.43 to 3.5 km. The basement surface beneath the study area appears to be disrupted by intrusive bodies. For example, profile P3–P3′ shows a basement uplift at ~ 0.43 km depth, whereas the basement deepens eastward to ~ 3.5 km along profile P1–P1′, reflecting the eastern sector of the area and the progressive deepening into the Bahariya Basin (Fig. 15). Greater basement depth may indicate higher groundwater potential, as deeper basins commonly accommodate thicker, more laterally extensive water-bearing sedimentary units. This finding is consistent with previous studies^30,76,82^.

**Table 3:** Description of 2D magnetic models.

Magnetic profile	Direction	Length (Km)	Magnetic reading (nT)	Basement depth (Km)
P1-P1`	E-W	80.10	−167 — −14	1.42 —3.50
P2-P2`	E-W	80.12	−133 — −24	0.95 — 2.84
P3-P3`	E-W	79.98	−80 — 102	0.43 — 2.48
P4-P4`	E-W	80.00	−114 — 105	0.98 — 2.93

### AHP-based weighting for groundwater potential assessment

**Fig. 16:**
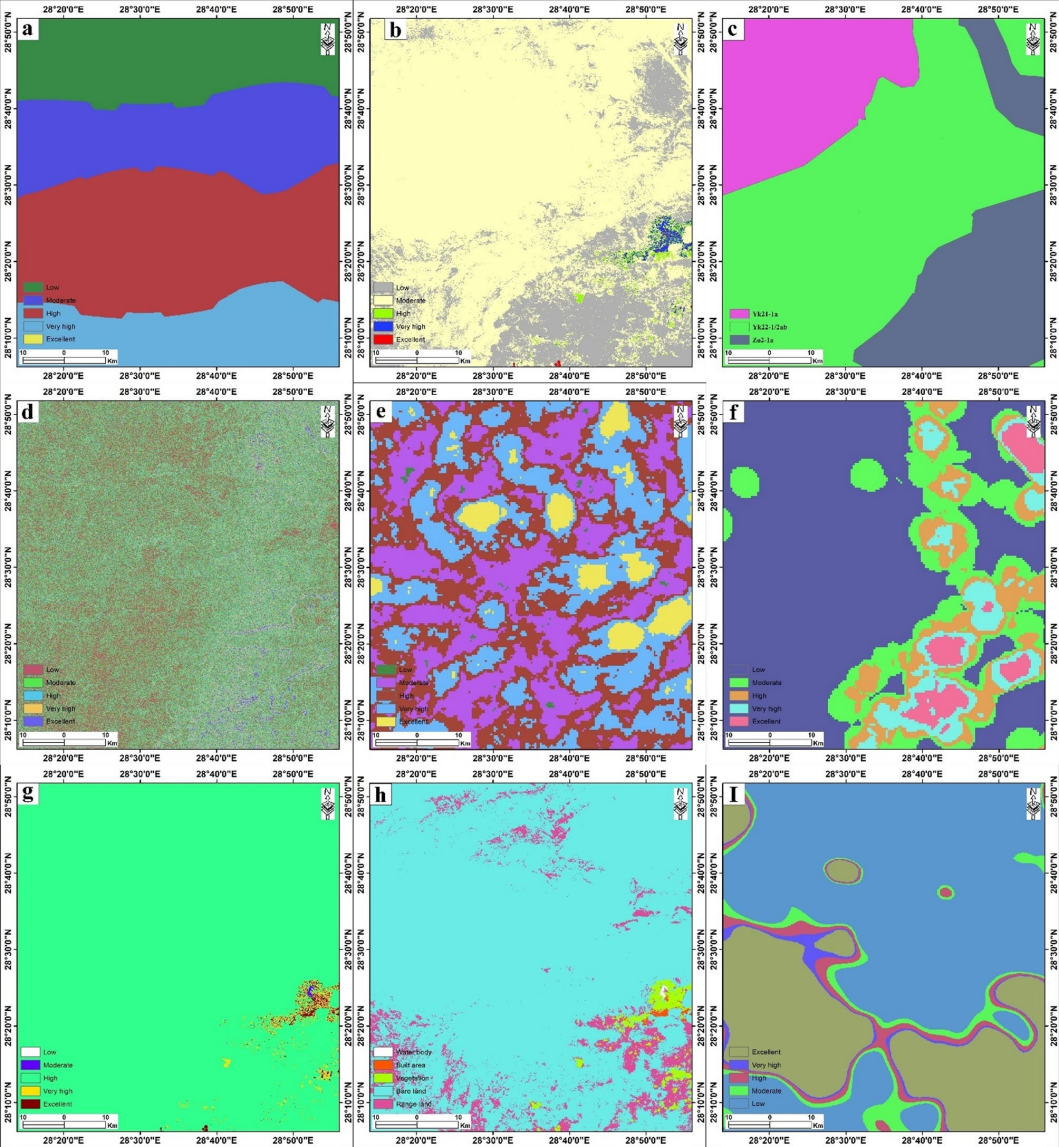
Reclassified thematic maps of (**a**) rainfall, (**b**) soil moisture, (**c**) Lithology, (**d**) slope, (**e**) drainage density, (**f**) Lineament density, (**g**) NDVI, (**h**) LU/LC, and (**i**) RTP of the study area. The figure was created by ArcGIS Desktop 10.8. (https://www.esri.com/enus/arcgis/products/arcgis-desktop/overview/).

To integrate diverse datasets into a single groundwater potential model, the thematic layers were reclassified using the Spatial Analyst extension in ArcGIS. The study utilized nine distinct variables: (a) Rainfall, (b) Soil Moisture, (c) Geology, (d) Slope, (e) Drainage Density, (f) Lineament Density, (g) NDVI, (h) Land Use/Land Cover (LU/LC), and (i) Reduced to Pole (RTP) magnetic data. To ensure spatial consistency, all layers were resampled to a uniform 30 m cell size. Each variable was then categorized into five potential zones based on its hydrogeological capability: Low, Moderate, High, Very High, and Excellent (Fig. 16). This reclassification facilitates a standardized arithmetic overlay, in which higher rank values correspond to zones with greater water-accumulation potential. Thematic map sub-classes were independently ranked on a scale of 0 to 9 based on their relative influence on groundwater recharge potential, with higher values assigned to classes demonstrating greater capacity for water recharge and storage (Table 4). The reclassified thematic layers were subsequently converted to raster format, resampled to the standardized 30-meter resolution, and georeferenced to the Universal Transverse Mercator (UTM) coordinate system to facilitate subsequent overlay and integration operations within the GIS environment.

**Table 4:** Saaty scale for pairwise comparison in the AHP technique.

Scales	Degree of preferences
1	Equally important
3	Moderately important
5	Strongly important
7	Very strongly important
9	Extremely important
2, 4, 6, 8	Intermediate values between the above judgments

The assignment of weights to individual thematic layers was performed using the Analytical Hierarchy Process (AHP) technique, a multi-criteria decision-making method developed by Saaty that provides a structured and transparent approach to integrating expert assessments with spatial data^9,10^. Rather than applying uniform weights across all parameters, a relative weighting scale ranging from 1 to 9 was systematically applied to each layer based on its respective significance and demonstrated capacity for water recharge. The weighting framework was informed by a comprehensive review of prior research conducted in hydrogeologically similar regions worldwide, complemented by field-based observations and expert judgment from regional geoscientists. This knowledge-based approach ensures that weights reflect the relative importance of each factor as established in the peer-reviewed literature while accounting for local hydrogeological conditions.

The AHP methodology proceeded through a series of pairwise comparisons among the nine thematic parameters, wherein each layer was systematically compared against every other layer using Saaty’s fundamental scale (1 to 9), with value definitions as follows: 1 (equal importance), 3 (moderate importance), 5 (strong importance), 7 (very strong importance), and 9 (extreme importance), with intermediate values (2, 4, 6, 8) allowing for gradual transitions between categorical distinctions (Table 4). These pairwise comparisons generated a normalized pairwise comparison matrix from which the Eigenvector approach derived the priority vector (PV) for each parameter. The resultant weights were normalized so that their sum equals unity (100%), ensuring an appropriate balance across the integrated model (Tables 5 and 6).

In the following, we will illustrate our rationale for each thematic layer parameter weight in the AHP model:

####  Primary controls (Highest weights)

- Rainfall (32.7%): In a hyper-arid environment, rainfall is the sole natural source of recharge. Its spatial distribution is therefore the first-order control on the possibility of groundwater replenishment, justifying its top priority. Therefore, it received the highest priority in pairwise comparisons (dominant over all other factors).
- Soil Moisture (17.5%): This is a direct indicator of near-surface water content. High soil moisture in arid zones strongly suggests recent infiltration, capillary rise from a shallow water table, or irrigation return flow, making it a highly relevant proxy for groundwater presence and potential recharge zones. It was judged to be very strongly important compared to most other factors.

#### Secondary terrain & Geological controls (Moderate weights)

- Slope (12.2%): Slope controls runoff velocity and infiltration time. Gentle slopes (0–5°) promote water stagnation and infiltration, while steeper slopes facilitate rapid runoff, reducing recharge potential. It is a fundamental terrain control on the recharge process. It was considered moderately to strongly more important than the geological/structural factors.
- RTP Aeromagnetic Data (8.76%): This layer provides insight into the subsurface structural framework and basement geometry, which controls deeper aquifer storage potential and the localization of groundwater along fault zones. Its weight is comparable to that of geology and drainage density, reflecting its importance in understanding the subsurface controls on groundwater accumulation.
- Geology (8.08%) & Drainage Density (8.08%): These were assigned equal, moderate weights based on their complementary roles. Geology (lithology) determines the inherent permeability and storage capacity of the surface materials. Drainage Density is an inverse proxy for infiltration; low-density areas indicate higher surface permeability where water infiltrates rather than being channeled away. Both were judged to be moderately important.

#### Indirect & Compound Indicators (Lower Weights)

Lineament Density (4.22%), NDVI (4.15%), and LU/LC (4.22%): These factors received lower but meaningful weights. Their rationale is as follows:

- Lineament Density (4.2%): A proxy for secondary (fracture) permeability. Its weight is balanced because not all lineaments are hydraulically conductive at depth.
- NDVI (4.2%): In arid regions where natural rainfall is insufficient, persistent healthy vegetation (high NDVI) often indicates phreatophytic behavior, access to groundwater. It is a valuable indirect bio-indicator of shallow groundwater availability, though it can also be strongly influenced by irrigation.
- LU/LC: Integrates human influence (irrigation via croplands) and natural conditions. It is highly correlated with soil moisture and NDVI, so its independent weight is appropriately lower to avoid double-counting effects.

To ensure the logical consistency and reliability of the weighting process, the consistency ratio (CR) was calculated from the pairwise comparison matrix. The CR serves as a quantitative measure of judgmental coherence, comparing the consistency index (CI) of the derived matrix against a random consistency index (RI) appropriate to the number of factors evaluated. A CR value below 0.10 (10%) indicates acceptable consistency and justifies acceptance of the assigned weights; conversely, CR values exceeding this threshold necessitate re-evaluation of pairwise judgments to achieve improved coherence. In this study, the CR is CR = 0.0045, indicating that the matrix is highly consistent (Table 6).

Although alternative multi-criteria evaluation techniques, including Fuzzy AHP and the Multi-Influencing Factor (MIF) method, are well-established in the literature, the conventional AHP was selected as the most appropriate methodology for this analysis. This selection reflects several methodological considerations: the availability of robust expert input from field campaigns and regional literature; the ordinal nature of the thematic layers and their varying influence on groundwater potential; and the specific research objectives, which emphasize transparent and reproducible decision-making within a GIS framework. Following weight normalization and consistency verification, the nine reclassified thematic layers were integrated using the Weighted Overlay Analysis (WOA) tool within ArcGIS Spatial Analyst. The integration formula follows:

$$GWPZ={\sum}_{i=1}^{n}({W}_{i}\times{R}_{i})$$

Where GWPZ represents the final groundwater potential zone index; ${W}_{i}$. denotes the normalized weight assigned to thematic layer i.; cap R sub i.. represents the reclassified rank value (0–9) for each cell within layer i.; and $n$ equals 9 (the total number of thematic layers). The weighted overlay operation produces a continuous raster surface reflecting the integrated influence of all groundwater-controlling factors, which is subsequently reclassified into the five potential zones corresponding to the output categories presented in Fig. 16.

**Table 5:** Rank, score, and weight assigned for the nine parameters.

Rank	Thematic layer	Class	Rank	Score
1	Rainfall	29.41–35.50	33	1
		35.51–41.6		2
		41.61–47.70		3
		47.71–53.80		4
		53.81–59.89		5
2	Soil moisture	−0.55- −0.09	18	1
		−0.09- −0.03		2
		−0.03- 0.11		3
		0.11–0.32		4
		0.32–0.83		5
3	Slope	0–3.10.10	12	1
		3.10–5.66		2
		5.66–8.94		3
		8.94–14.23		4
		14.23–46.53		5
4	Geology	Yk21	8	1
		Yk22		3
		Zo		5
5	Drainage density	171.34–254.78.34.78	8	5
		140.40–171.33.40.33		4
		117.89–140.39.89.39		3
		86.941–117.88		2
		15.68–86.94		1
6	Lineament density	0–6.9383.9383	4	1
		6.94–13.88		2
		13.88–20.82		3
		20.82–27.75		4
		27.75–34.69		5
7	NDVI	0.34–0.91	4	5
		0.14–0.34		4
		0.04–0.14		3
		−0.14-0.04		2
		−1.0- −0.14		1
8	LU/LC	Bare lands	4	1
		Range lands		2
		Built areas		3
		Vegetation		4
		Water body		5
9	RTP	< (−102)	9	5
		(−102) - (−75)		4
		(−75) – (−46)		3
		(−46) – (20)		2
		> 20		1

**Table 6:** Pairwise comparison matrix for the nine parameters.

Parameter	Rainfall	Soil	Slope	Geology	Drainage d.	Lineament d.	NDVI	LU/LC	RTP	Normalized Principal Eigenvector
Rainfall	1	2	3	4	4	7	8	7	4	32.74%
Soil	1/2	1	2	2	2	4	4	4	2	17.51%
Slope	1/3	1/2	1	2	2	3	3	3	1	12.23%
Geology	1/4	1/2	1/2	1	1	2	2	2	1	8.08%
Drainage density	1/4	1/2	1/2	1	1	2	2	2	1	8.08%
Lineament density	1/7	1/4	1/3	1/2	1/2	1	1	1	1/2	4.22%
NDVI	1/8	1/4	1/3	1/2	1/2	1	1	1	1/2	4.15%
LU/LC	1/7	1/4	1/3	1/2	1/2	1	1	1	1/2	4.22%
RTP	1/4	1/2	1	1	1	2	2	2	1	8.76%

Consistency Index (CI) ≈ 0.0066.

Consistency Ratio (CR) ≈ 0.0045.

### Groundwater potential mapping of Bahariya Oasis

**Fig. 17:**
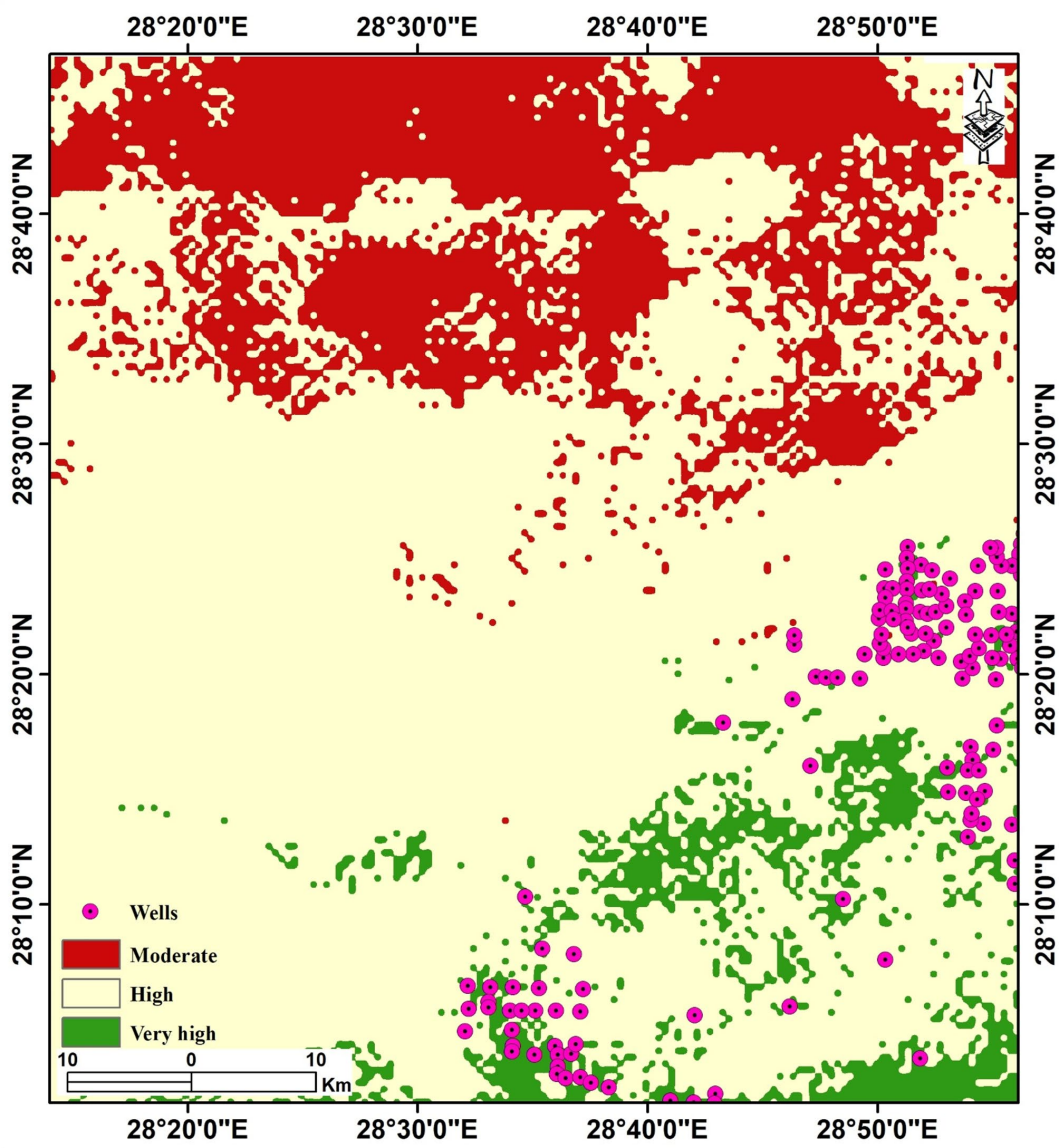
A groundwater potential map was constructed by integrating nine remote sensing and geophysical parameters. The figure was created by ArcGIS Desktop 10.8. (https://www.esri.com/enus/arcgis/products/arcgis-desktop/overview/).

The integrated Analytical Hierarchy Process (AHP) model, which combines nine weighted thematic layers of hydrogeological, structural, and morphometric characteristics, was applied to the entire Bahariya study area to generate a comprehensive groundwater potential map (Fig. 17). The results delineate three primary groundwater potential zones: Very High, High, and Moderate, with the distribution reflecting the underlying geological, hydrological, and structural architecture of the depression.

#### Spatial distribution of groundwater potential

The groundwater potential map reveals pronounced spatial heterogeneity across the depression. Very High potential zones (depicted in dark green) are concentrated predominantly in the southern and southeastern portions of the depression, corresponding to areas with high lineament density, favorable slopes, permeable Quaternary alluvial deposits, and elevated soil moisture (Figs. 4b, 5b and 17). These zones align closely with the major wadi outlets where flash flood water concentrates during precipitation events, facilitating infiltration into the shallow aquifer.

High potential zones (light green) are scattered throughout the central depression and along the margins of major drainage channels, reflecting moderate to good combinations of hydrogeological parameters. These areas remain suitable for groundwater development, particularly where access to the water table is feasible with shallow drilling (Fig. 17). Moderate potential zones (beige/tan) occupy the western margins and portions of the northern depression floor, where either steeper topography, thicker clay confining layers, or lower lineament density reduces groundwater accessibility. On the other hand, the northern and plateau regions display predominantly Moderate to Low potential, dominated by dense limestone with minimal fracturing, and by high-altitude locations that limit direct recharge (Fig. 17).

Notably, the distribution of groundwater potential zones closely corresponds to the drainage network analyzed in the previous section on the DEM. The concentration of Very High potential areas in the south aligns with the terminus of major wadis, where runoff accumulates and infiltrates. This spatial concordance validates the multi-parameter approach and reinforces the conceptual model of Bahariya as a closed basin with centripetal flow toward accumulation and recharge zones.

### Validation of the groundwater potential model

To rigorously evaluate the predictive accuracy of the AHP-derived groundwater potential map, a Receiver Operating Characteristic (ROC) and Area Under the Curve (AUC) validation was conducted. The ROC-AUC method is considered among the most reliable and statistically robust approaches for assessing model performance in spatial predictive modeling^10,11,76,77,84,85^. The final integrated groundwater potential map was validated and its predictive accuracy quantitatively assessed using the ROC and AUC analysis. This validation was performed using a set of 193 known well locations within the study area, providing a statistically robust measure of the model’s ability to distinguish between productive and non-productive zones.

#### ROC-AUC results

**Fig. 18:**
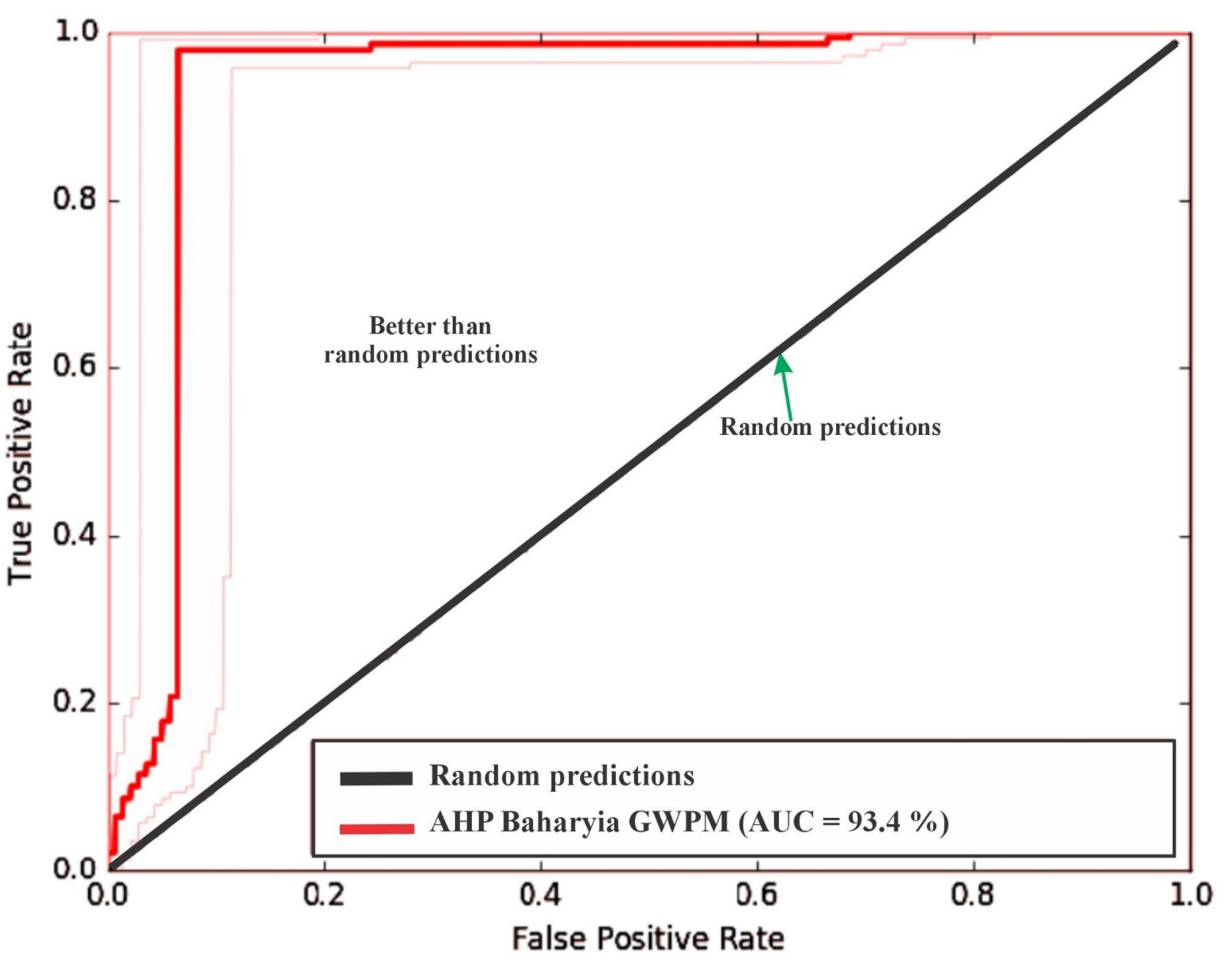
AUC curve of the GWPM in the Bahariya area. The figure was created by ArcGIS Desktop 10.8. (https://www.esri.com/enus/arcgis/products/arcgis-desktop/overview/).

The ROC curve analysis yielded an Area Under the Curve (AUC) value of 93.4%, as shown in Fig. 18. This exceptionally high AUC value indicates that the AHP model demonstrates excellent predictive accuracy, substantially outperforming random prediction (which would yield an AUC of 50%). The steep rise in the ROC curve near the origin reflects the model’s strong discriminatory power, as the majority of actual groundwater zones are correctly classified with minimal false positives.

#### Model interpretation and reliability

An AUC of 93.4% signifies that, when a randomly selected point from a groundwater-rich zone is compared to a randomly selected point from a groundwater-poor zone, the model correctly ranks the groundwater-rich point as having higher potential in 93.4% of comparisons (Fig. 18). This performance level provides strong confidence that the delineated Very High and High potential zones are genuinely favorable for groundwater development.

#### Implications for water resource management

The high model accuracy validates the utility of integrating remote sensing, GIS, aeromagnetic data, and multi-criteria decision analysis for groundwater prospecting in arid regions. The Bahariya groundwater potential map provides a robust basis for sustainable well siting, infrastructure planning, and water security assessments. For regions such as Bahariya, where groundwater is the sole renewable source for agricultural productivity and human settlement, this model-derived guidance is critical for managing water scarcity and supporting integrated resource development.

## Discussion

The integrated GIS-based Analytical Hierarchy Process (AHP) model, combining multi-source remote sensing thematic layers with reduced-to-pole (RTP) aeromagnetic data, provides a robust delineation of groundwater potential zones in the Bahariya Oasis, a key arid depression within Egypt’s Western Desert and the Nubian Sandstone Aquifer System (NSAS). To evaluate the robustness of the Analytical Hierarchy Process (AHP) model and the relative contribution of each thematic layer, a sensitivity analysis was performed by systematically adjusting the assigned weights. In this approach, the weights of the primary predictors Rainfall (32.74%), Soil Moisture (17.51%), and Slope (12.23%) (Tables 5 and 6; Supplementary Fig. 3) were varied by ± 10% to assess the stability of the final Groundwater Potential Zone (GWPZ) index. The results revealed that the spatial extent of the “Very High” potential zones remained remarkably consistent, with a total area variation of only 4.5%, confirming that the model is robust and not overly sensitive to individual weighting subjectivities (Fig. 17). The analysis further indicated that integrating RTP aeromagnetic data (8.76%) and Lineament Density (4.22%) (Tables 5 and 6; Supplementary Fig. 3) provided unique subsurface structural constraints that surface-only models lack, thereby enhancing the overall reliability of the GWPZ classification (Fig. 17). This is consistence with the previous works, which recommended integrated remote sensing with geophysical data to enhance the accuracy of the outcomes^9,10,17^, and can assist decision-makers in planning effective groundwater extraction strategies. The resulting map identifies coherent “very high” to “high” potential corridors predominantly in the southern and southeastern sectors, where favorable surface and subsurface conditions converge (Fig. 17). This spatial pattern aligns closely with known productive well distributions and hydrogeological features, yielding an excellent ROC-AUC of 93.4% (Fig. 18) on an independent validation subset (193 wells), confirming strong discriminatory performance. The findings highlight the utility of integrating remote-sensing-based GIS and aeromagnetic data using the AHP. This synergistic approach provides access to advanced analytical capabilities for studying and managing groundwater resources^9–11,86–90^. Remote sensing-derived proxies effectively capture surface controls on recharge and infiltration. High lineament density, extracted from Sentinel-1 data (Fig. 4), serves as a reliable indicator of fracture networks that enhance permeability and preferential recharge pathways in the arid setting. This is particularly evident in the southern oasis margins, where dense lineaments coincide with low-relief topography (gentle slopes < 5°) that minimizes runoff and promotes infiltration (Fig. 5b). Drainage density further support recharge potential, as higher values in Quaternary alluvial-filled depressions facilitate downward percolation, albeit moderated by irrigation return flow dominance over limited rainfall (Fig. 6c). Land-surface indicators NDVI and soil moisture from Sentinel-2 (Fig. 2b, c) highlight vegetated/moist zones in cultivated areas, acting as proxies for localized recharge and aquifer vulnerability. In contrast, permeable lithologies (e.g., Quaternary sands/alluvium) and favorable LULC (agricultural/oasis deposits) reinforce accumulation in low-lying sectors (Fig. 2d). These surface proxies, weighted via AHP with rainfall at 32.7% (Fig. 2a; Table 6), reflecting potential recharge contribution despite aridity, collectively drive the model’s surface expression of potential.

Aeromagnetic data provide critical subsurface constraints, resolving basement configuration and structural architecture not accessible via remote sensing alone. RTP processing and interpretation reveal marked basement deepening toward the south and southeast, with magnetic lows indicating thicker sedimentary cover (enhanced storage capacity in Nubian aquifer sequences S1–S3)^74–77^. The upward-continuation results provide insight into the depth distribution and structural organization of the magnetic sources underlying the study area^38,39^. The persistence of a central magnetic high across all continuation levels indicates a deeply rooted basement high or intrusive body. In contrast, the increasing coherence of surrounding magnetic lows, particularly around the Bahariya-1 well, delineates a regionally extensive, sediment-filled basement depression, together defining a segmented basement architecture that fundamentally controls aquifer thickness and groundwater storage potential^30^. Upward continuation is applied as a qualitative filter to evaluate relative depth contributions rather than as a unique depth estimator^39^, and, when integrated with Euler deconvolution and 2D magnetic modeling, it delineates a clear contrast between uplifted basement blocks and deep sedimentary depressions, providing a depth-aware structural framework that strengthens groundwater potential mapping in basement-controlled arid aquifer systems^10,11,21^. Qualitative interpretation of RTP and upward-continued magnetic maps can be ambiguous and non-unique^36,65^, necessitating the application of advanced signal-processing techniques. Accordingly, the TAHG and MGTHG edge-detection filters were applied to the RTP and UC data to enhance the delineation of both shallow and deep magnetic boundaries, as these filters provide higher-resolution imaging than conventional methods and effectively delineate faults, fractures, lithological contacts, and subtle magnetic contrasts that govern groundwater flow and storage^50,66–68^.

The TAHG and MGTHG-RTP datasets, along with their upward-continued (1–5 km) maps (Figs. 7 and 8), delineate dominant ENE–WSW, NE–SW, and NW–SE magnetic lineaments that are consistent with the regional fault framework and are interpreted as preferential pathways for groundwater flow. Progressive smoothing of anomalies with increasing continuation height indicates predominantly shallow sources related to fractured or weathered basement, while persistent linear features near the Bahariya-1 and HF36-4 wells suggest deeper fault systems that may act as recharge conduits. The mapped orientations agree with established tectonic models for Bahariya Oasis^3,71–74^, including Syrian Arc–related deformation and later NW-trending Tertiary faults, and are consistent with Landsat-derived structures (Fig. 4). The increasing prominence of NW–SE trends at greater depths further reflects older Paleozoic–Cretaceous structural fabrics^50,75–77^, underscoring the influence of multiple tectonic phases on groundwater-controlling fracture systems^10,30,74^. From a hydrogeophysical perspective, the superposition of structural systems strongly influences groundwater occurrence and flow, with intersecting fault and fracture networks enhancing secondary porosity and hydraulic connectivity, while depth-persistent NW–SE lineaments locally control aquifer thickness and storage. The consistency between magnetic-derived structures and Landsat lineaments confirms the robustness of this framework and demonstrates the value of aeromagnetic analysis in providing depth-aware constraints essential for groundwater assessment in basement-controlled arid regions. The correspondence between high lineament density (Fig. 13) and regional structural trends reflects multiple deformation phases^30,75,79–81^ and highlights structurally controlled zones with enhanced potential for subsurface fluid flow.

The Euler deconvolution results provide complementary depth constraints, showing shallow to intermediate solutions associated with near-basement structures and deeper NW–SE solutions indicative of vertically extensive fault zones. Their correspondence with CET-derived lineaments confirms a structurally segmented basement, consistent with earlier interpretations^30,50,64,75–81^, and highlights fault architectures that control aquifer thickness, hydraulic connectivity, and regional groundwater flow. The 2D magnetic modeling provides depth-resolved constraints on basement relief, revealing a structurally segmented basement of uplifted blocks and deeper depressions that exerts first-order control on sediment thickness and aquifer geometry in arid basement settings. Basement depths range from ~ 0.43 to 3.5 km (Fig. 15; Table 3), with a shallow uplift along profile P3–P3′ and progressive eastward deepening to ~ 3.5 km along P1–P1′ toward the Bahariya Basin, conditions that favor thicker sedimentary successions and enhanced groundwater potential, consistent with earlier studies^30,76,77,82^. Although constrained by limited borehole control and inherent non-uniqueness, integration with Euler depth solutions and aeromagnetic lineament analysis reduces ambiguity and demonstrates the value of a depth-aware aeromagnetic framework for groundwater assessment in data-scarce arid regions.

Overall, the integrated aeromagnetic analysis confirms that basement architecture is a primary control on groundwater systems, with basement depth regulating aquifer thickness and storage, and magnetic lineament patterns reflecting fault-controlled secondary porosity and transmissivity. Deep basement depressions with high structural complexity favor groundwater accumulation and flow, whereas uplifted or fault-bounded blocks may compartmentalize the aquifer, underscoring the value of aeromagnetic data for depth-aware groundwater assessment in basement-influenced arid regions.

The integration between datasets is evident in the spatial convergence of indicators. For instance, surface lineament clusters and drainage networks closely follow aeromagnetic-derived structural trends, implying tectonic control on both infiltration pathways and subsurface flow. In the southern/southeastern sectors, the combination of high lineament density, low relief, permeable Quaternary deposits, and deeper basement results in optimal conditions for storage and recharge, forming coherent potential zones that track oasis drainage patterns. These findings have direct implications for water security in Bahariya, a priority area for Egyptian agricultural and developmental initiatives. “Very high” and “high” zones offer prime targets for prioritized drilling, potentially reducing empirical siting risks and improving success rates in the non-renewable NSAS. However, correlations between thematic layers and potential zones are indicative rather than causal, reflecting proxy-based favorability amid complex hydrogeological interactions (e.g., irrigation dominance, historical drawdown).

In this study, model uncertainty was assessed by quantitatively validating the AHP-derived output against 193 independent well occurrences using ROC-AUC analysis (Fig. 17). The resulting AUC of 93.4% demonstrates excellent predictive accuracy (Fig. 18). These results are consistent with similar studies^90^. The findings highlight the utility of integrating remote-sensing-based GIS and aeromagnetic data using the AHP. This synergistic approach provides access to advanced analytical capabilities for studying and managing groundwater resources^9–11,86–90^. The model is subject to inherent uncertainties stemming from the semi-subjective nature of the pairwise comparison matrix and the indirect nature of proxies such as NDVI and Soil Moisture. These indicators can be influenced by anthropogenic factors, such as irrigation return flow, which may obscure natural recharge signals. Additionally, the use of regional-scale aeromagnetic data introduces a degree of “lithologic masking,” where shallow Tertiary volcanic intrusions may interfere with deep basement interpretations; however, this was mitigated through upward continuation and TAHG/MGTHG filtering. While the high AUC indicates strong discrimination, it does not fully capture subsurface heterogeneity or long-term productivity. The model’s ability to identify areas where groundwater is accessed constitutes a limited form of ground truth. It does not validate predicted yields, water quality, or sustainable abstraction rates. The most significant uncertainty stems from the lack of hydraulic data (e.g., transmissivity, specific capacity) for quantitative correlation with the potential index and comparison with pumping test data, groundwater depth, or yield data. The model is therefore best used as a regional-scale targeting tool to prioritize areas for subsequent, detailed hydrogeological investigations that can acquire such ground-truth data.

The AHP model integrates multiple thematic layers as proxies for hydrogeological favorability rather than direct causal drivers. For instance, high lineament density is interpreted as an indicator of potential fracture-controlled permeability and enhanced recharge pathways, while magnetic lows (deeper basement) correlate with thicker sedimentary cover and greater storage capacity. These relationships are supported by spatial overlap with well data and regional hydrogeological understanding but do not establish unidirectional causation, as groundwater occurrence is influenced by interconnected factors, including historical abstraction, irrigation, and unmodeled subsurface heterogeneity.

Future work should focus on enhancing the model’s certainty and practical utility through several ways: (i) integrating direct hydraulic data (e.g., well yield, transmissivity) where available to move beyond positional validation towards quantitative resource validation; (ii) developing spatially explicit uncertainty maps by propagating errors from source data and AHP weights, potentially using Monte Carlo simulation; and (iii) applying the established integrated workflow (remote sensing, GIS-AHP, aeromagnetics) to other arid basins to test its transferability and refine the selection and weighting of thematic layers for different hydrogeological settings. These steps will improve the interpretability of GWPZ maps, support risk-informed decision-making for drilling and managed aquifer recharge, and strengthen the framework for sustainable groundwater exploration in data-scarce regions.

## Conclusions

- Groundwater potential in Bahariya is primarily influenced by the depression’s closed-basin shape, centripetal drainage patterns, and NE-SW trending fault systems, which promote the formation of secondary aquifers. The highest groundwater potential areas are found in the southern and southeastern parts of the study area, where gentle slopes, high lineament density, and permeable Quaternary deposits enhance infiltration and groundwater availability.
- The Analytical Hierarchy Process successfully combined nine thematic layers (rainfall, soil moisture, geology, slope, drainage density, lineament density, NDVI, LU/LC, and RTP) to produce a groundwater potential map with 93.4% validation accuracy (AUC), showing that multi-parameter modeling is more effective than single-factor methods for mapping groundwater in complex arid systems.
- Aeromagnetic interpretation clarified the subsurface structural framework and constrained the basement geometry, revealing pronounced deepening of the basement toward the southern and southeastern sectors. The thick sedimentary cover in these areas implies enhanced groundwater storage potential and independently supports the groundwater potential zoning results. In addition, the magnetic data delineate dominant ENE–WSW, NE–SW, and NW–SE lineament sets, consistent with regional fault systems that likely act as preferential pathways for groundwater movement.
- The high-accuracy groundwater potential map is a valuable tool for guiding well development, improving exploration efficiency, and planning water infrastructure in Bahariya Oasis. This combined approach, utilizing remote sensing, GIS, and aeromagnetic data, can be applied to other arid basins, providing a replicable framework for sustainable groundwater management in water-scarce areas.
- We recommend that future works depend on integrated methods and additional ground data (e.g., borehole geophysical logs or yield measurements) to strengthen validation and enhance AHP limitations.

## Supplementary Information

Below is the link to the electronic supplementary material.

Supplementary Material 1

## Data Availability

The datasets used and/or analyzed during the current study are available from the corresponding author upon reasonable request.
