# Supplementary material for: Mapping and predicting groundwater accumulations using remote sensing and aeromagnetic data: a case study from Bahariya Oasis, Western Desert, Egypt
Source: Sci Rep. 2026 Mar 26;16:10489. doi: 10.1038/s41598-026-42907-z (PMC13031932; doi:10.1038/s41598-026-42907-z)
Supplement: Supplementary file 1 — Supplementary Material 1 [file 41598_2026_42907_MOESM1_ESM.docx]

**Materials and Methods**

***Considerations on Data Temporal Compatibility***

The datasets used in this study were acquired over different periods: aeromagnetic surveys (1980s), the SRTM DEM, and Sentinel-1/2 imagery (2021). The temporal compatibility for groundwater potential mapping is justified based on the characteristic timescales of the geological and geomorphological processes being analyzed:

1. ***Geological Structure & Basement Geometry (Aeromagnetic Data):*** The configuration of the Precambrian basement and the major fault systems that control basin architecture evolve over millions of years. Therefore, data from the 1980s accurately represent the current deep structural framework, as no significant tectonic activity has occurred in the intervening decades.
2. ***Regional Topography & Drainage Network (SRTM DEM):*** The regional-scale morphology of the Bahariya depression, its escarpments, and the primary drainage patterns are geomorphologically stable over century to millennial timescales. The SRTM DEM, therefore, provides a valid and temporally consistent representation of the topographic controls on surface runoff and recharge pathways.
3. ***Surface Conditions & Vegetation (Sentinel-1/2):*** These datasets provide a snapshot of surface conditions (soil moisture, NDVI, LULC) for the year 2021. While these parameters can exhibit seasonal and inter-annual variability, the 2021 data are considered representative of the recent average state of the oasis. For the purpose of identifying *potential* zones, the spatial *patterns* (e.g., the concentration of vegetation and moisture within the central depression versus the barren plateaus) are more critical than absolute values from a specific date, as these patterns are persistent features of the irrigated oasis system.

**Aeromagnetic data processing**

**High-precision edge detection**

Edge detection of magnetic sources is vital in interpreting subsurface geological features from magnetic data. In recent years, numerous edge detection techniques have been widely utilized to enhance the visibility of geological structures such as faults, lithological contacts, and dykes [41-42]. Geologically, these edges represent contacts delineating zones of similar rock types [43]. The advancement of high-resolution magnetic filtering methods has significantly improved the accuracy of structural mapping. This study selected and applied two refined edge detection techniques to the magnetic dataset to accurately delineate magnetic boundaries and support the geological interpretation:

1. *The tilt angle of the Horizontal Gradient (TAHG)*

The TAHG filter is an edge detection technique designed to enhance magnetic anomalies. This method utilizes the tilt angle of the total horizontal gradient to highlight geological boundaries. One of its key advantages is its ability to generate amplitude maxima directly above the edges of magnetic sources. Additionally, it effectively balances the signal response from both shallow and deep sources, making it particularly useful for mapping structural features at varying depths with consistent clarity [35]. Due to the arctangent function's properties, the TAHG transform's output range is confined to −π/2 to +π/2 radians. This bounded range ensures the filter provides stable, interpretable results, allowing consistent detection of source edges regardless of the depth or magnitude of the magnetic anomaly. The corresponding equation is expressed as:

$TAHG= atan \left( \frac{{HGA}_{Z}}{\sqrt{{{HGA}_{X}}^{2}+{{HGA}_{Y}}^{2}}} \right)$ (1)

1. *Modified Gudermannian function of total horizontal gradient (MGTHG)*

The MGTHG filter, recently developed by [36], incorporates the Modified Gradient Filter (MGF) proposed by [44] to enhance the delineation of horizontal boundaries associated with potential-field anomalies at varying depths. This advanced filtering technique increases edge detection accuracy by enhancing the resolution of potential field data, enabling more precise structural interpretations. The MGTHG filter is mathematically defined as follows:

$MGTHG= \frac{2}{\pi} atan \left\{ sinh\left( \frac{{HGA}_{Z}+{HGA}_{Z}-\sqrt{{{HGA}_{X}}^{2}+{{HGA}_{Y}}^{2}}}{\sqrt{{{HGA}_{X}}^{2}+{{HGA}_{Y}}^{2}}} \right) \right\}$ (2)

The peaks of the MGTHG filter effectively delineate the edges of causative geological bodies. Its amplitude ranges from –1 to +1 radians, providing a normalized scale for interpretation. A key advantage of the MGTHG filter is its ability to delineate structural boundaries with high precision and clarity. In contrast to many contemporary high-resolution edge detection techniques, the MGTHG filter delivers consistent resolution in its outputs, independent of user-defined parameters [45-47].

**Center of exploration and targeting grid analysis technique (CET)**

The Center for Exploration Targeting–Grid Analysis (CET) technique is a powerful tool for enhancing magnetic image textures, enabling the identification of structurally complex zones that may be significant for groundwater exploration. The Structural Complexity (SC) approach, introduced by [48], is particularly effective in identifying structurally complex zones that may influence groundwater flow and accumulation, making it valuable for locating areas with high groundwater potential. CET employs a systematic analytical workflow that evaluates image texture to reveal critical structural elements such as contacts, edges, and boundaries.

The process begins by calculating the standard deviation of the magnetic data to assess spatial variability. This is followed by phase symmetry analysis to highlight ridge-like features in the dataset. An amplitude thresholding step is then applied to isolate these ridges, after which a skeletonization process reduces them to their central axial lines. In the final step, the extracted texture peaks from the phase symmetry are used to generate vectorized linear features. Magnetic anomalies identified through this method often correspond to geological structures such as faults, lithological contacts, and dykes. These interpreted features contribute significantly to understanding the geological framework at both regional and local scales and assist in targeting areas with favorable conditions for groundwater exploration [49-50].

**Depth estimation method**

- **3D-Euler depth estimation method (EUD)**

Euler Deconvolution (EUD) was initially introduced by [51] as an automated approach for determining the location and depth of magnetic sources using realistic magnetic profiles and datasets. This method was later adapted by [52] to work effectively with gridded magnetic data. Euler deconvolution estimates the depth of subsurface sources by applying spatial derivatives of potential field data [53]. It is widely employed to delineate subsurface structural boundaries and to identify fault trends. The underlying principle is based on the homogeneity relationship, which can be mathematically expressed as follows:

∂T∕∂­x (x − x_0_)­+ ∂T∕­∂y (y − y_0_)­ + ∂T∕­∂z (z − z_0_)­ = SI (B − T) (3)

The Structural Index (SI) characterizes the geometry of subsurface magnetic sources, while B represents the regional or base level of the magnetic field, and T denotes the measured magnetic field at specific spatial coordinates (x, y, z). In this study, the Euler deconvolution method was applied to the reduced-to-pole (RTP) magnetic dataset to identify structural features that may control groundwater movement, such as faults and fracture zones, and to estimate the depths of their associated magnetic sources. This information is crucial for understanding subsurface conditions favorable for groundwater accumulation.

- **Modelling**

Modelling represents the final phase in the interpretation of geophysical data and should incorporate all relevant, high-quality information to constrain the results [54]. This process combines data from multiple sources to estimate spatial variations in physical properties, such as magnetic susceptibility. In this study, five two-dimensional (2D) magnetic models were developed along selected profiles using the GM-SYS modeling software [55]. These profiles were carefully chosen to cover most of the study area and to intersect the two existing boreholes (Fig. 6a), ensuring that both regional and local geological features were captured. Magnetic susceptibility values used in the modeling were taken from established references, including [56-58]. In general, sedimentary rocks exhibit significantly lower magnetic susceptibility than igneous rocks and are often considered to have near-zero values in regional-scale interpretations [59].


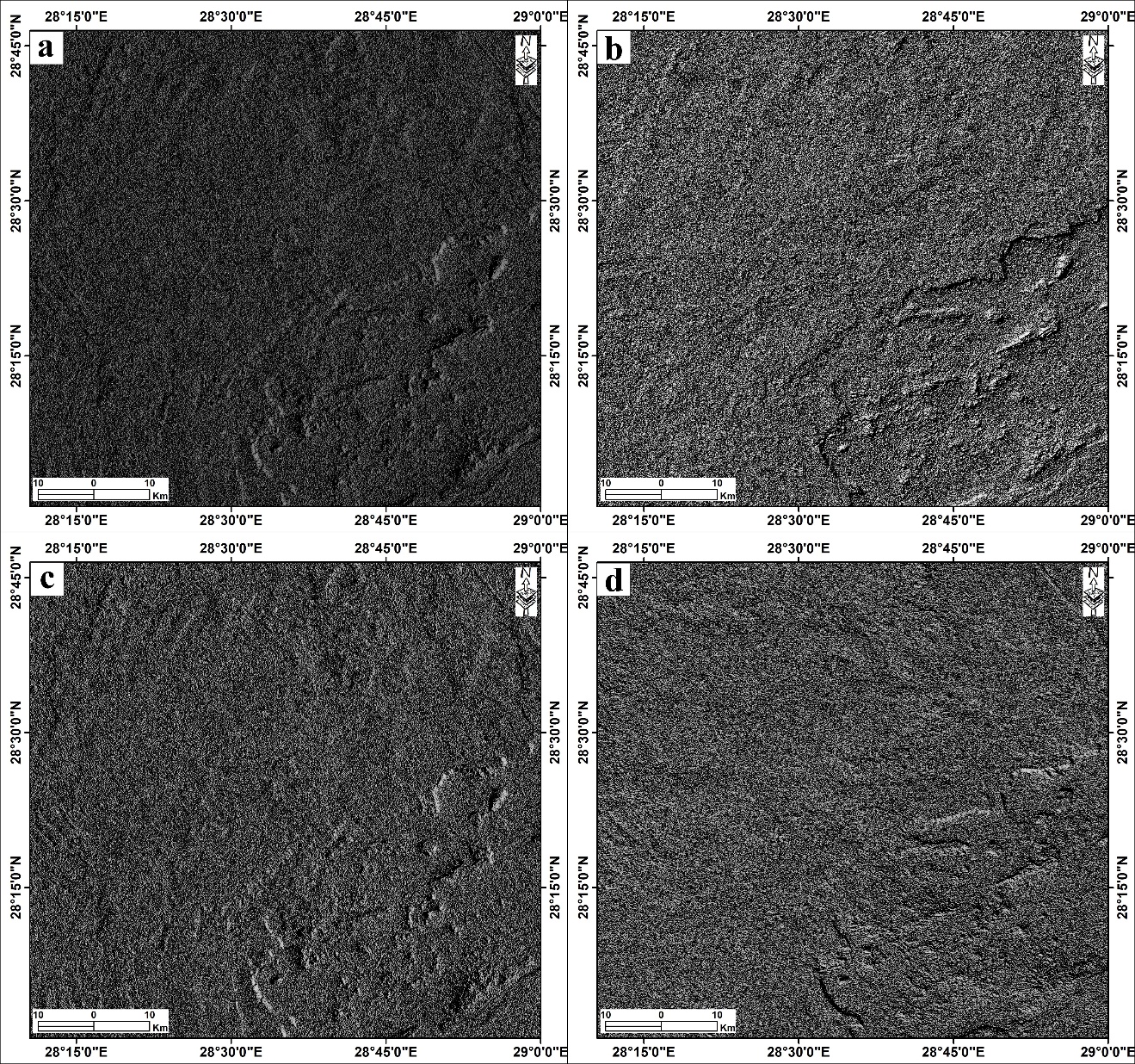


**Supplementary Fig. 1.** SRTM-DEM Shaded-relief image derived using a light source azimuth in different angles and an altitude of 45° (A) Angle 0°. (B) 45°. (C) 90°. (D) 135°.The figure was created by ArcGIS Desktop 10.8. (<https://www.esri.com/enus/arcgis/products/arcgis-desktop/overview/>).


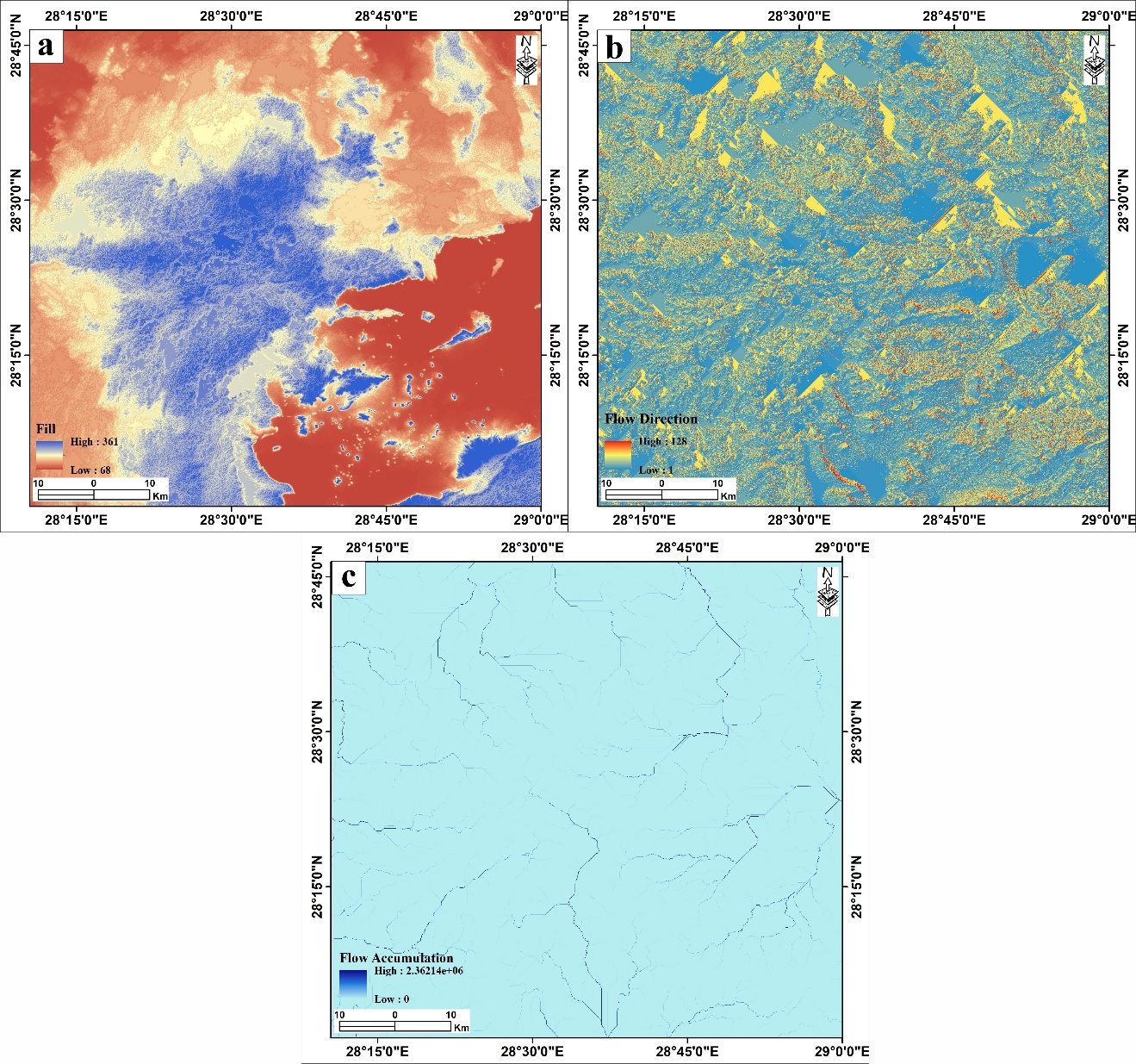


**Supplementary Fig.2.** Hydrological analysis of the Baharyia area. (a) Fill, (b) Flow Direction, and (c) Flow Accumulation. The figure was created by ArcGIS Desktop 10.8. (<https://www.esri.com/enus/arcgis/products/arcgis-desktop/overview/>).

**Supplementary Table 1.** AHP weights and sensitivity (Error bar) for the selected thematic layers

| Thematic Layer | Final Weight (%) | Sensitivity (Error Bar ±) | Rationale for Weighting |
| --- | --- | --- | --- |
| Rainfall | 32.74% | 3.20% | Main source of contemporary recharge. |
| Soil Moisture | 17.51% | 4.80% | Critical proxy for shallow water tables. |
| Slope | 12.23% | 2.10% | Controls infiltration-to-runoff ratio. |
| RTP Magnetic | 8.76% | 1.50% | Defines the subsurface structural basin. |
| Geology | 8.08% | 1.20% | Controls the primary permeability of units. |
| Drainage Density | 8.08% | 1.80% | Indicates surface runoff efficiency. |
| Lineament Density | 4.22% | 0.90% | Proxy for structural secondary porosity. |
| LULC | 4.22% | 0.50% | Identifies current groundwater usage. |
| NDVI | 4.15% | 0.40% | Bio-indicator of accessible moisture. |


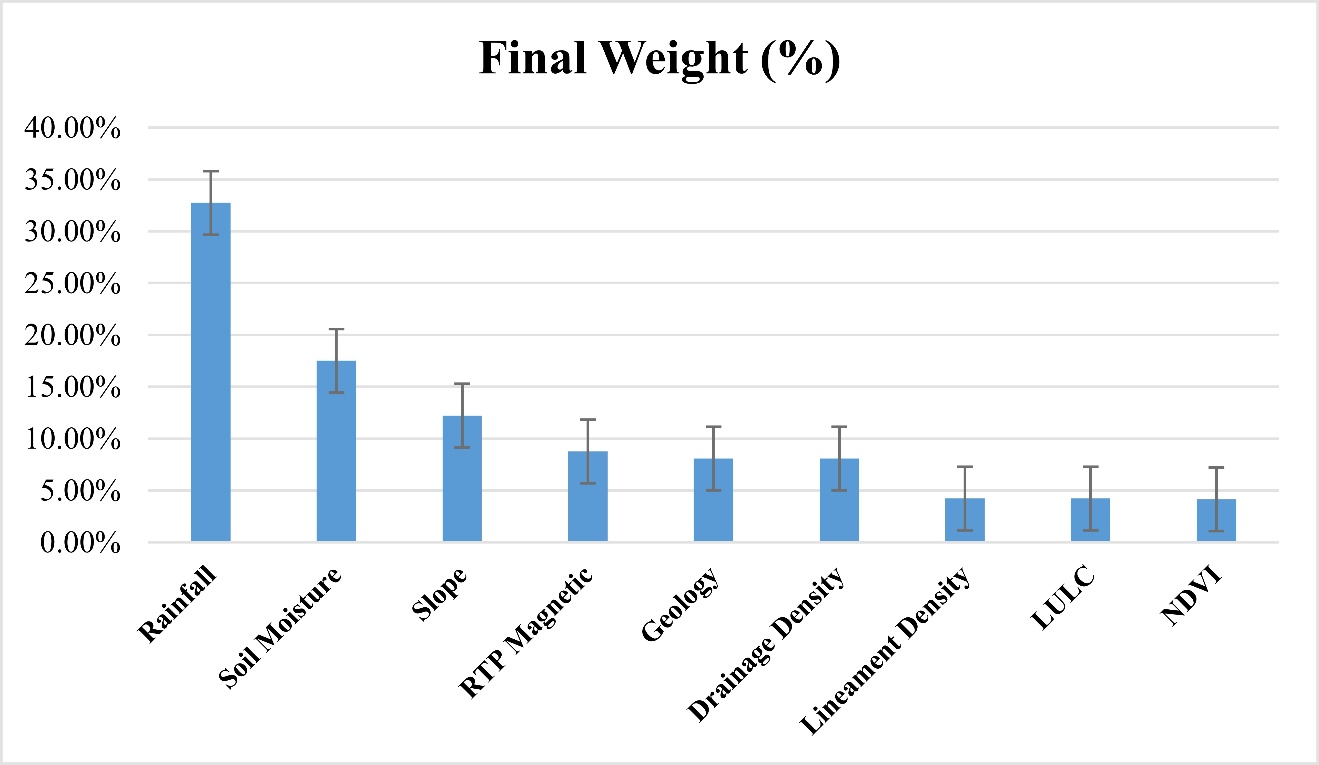


**Supplementary Fig. 3.** The weights of the thematic layers were normalized to create the groundwater potential map.
